# Supplementary material for: Climate response to Nature Future scenarios in a regional Earth System Model
Source: Nat Commun. 2026 Mar 16;17:4017. doi: 10.1038/s41467-026-70284-8 (PMC13136345; doi:10.1038/s41467-026-70284-8)
Supplement: Supplementary file 1 — Supplementary Information [file 41467_2026_70284_MOESM1_ESM.pdf]

Supplementary information

Climate response to Nature Future scenarios in a regional Earth System Model

Petra Sieber[1,*], Dirk Nikolaus Karger[2], Niklaus E. Zimmermann[2], Sara Si-Moussi[3], Wilfried Thuiller[3], Gabriele Midolo[4], Milan Chytrý[5], Irena Axmanová[5], Jonas Schwaab[1,6,7], Edouard L. Davin[8,9,10], Matthieu Leclair[11], Stephan Kambach[12], Helge Bruelheide[12,13], Thomas Hickler[14,15], Zvjezdana Stančić[16], Idoia Biurrun[17], Behlül Güler[5,18], Jürgen Dengler[19,20], Jan Divíšek[5], Peter H. Verburg[21], Sonia I. Seneviratne[1]

[1]Institute for Atmospheric and Climate Science, ETH Zurich, Zurich, Switzerland
[2]Swiss Federal Institute for Forest, Snow and Landscape Research WSL, Birmensdorf, Switzerland
[3]University of Grenoble Alpes, University of Savoie Mont Blanc, CNRS, LECA, Grenoble, France
[4]Department of Spatial Sciences, Faculty of Environmental Sciences, Czech University of Life Sciences Prague, Praha-Suchdol, Czech Republic
[5]Department of Botany and Zoology, Faculty of Science, Masaryk University, Brno, Czech Republic
[6]Institute for Environmental Planning, Leibniz Universität Hannover, Hannover, Germany
[7]Alpine Environment and Natural Hazards, WSL Institute for Snow and Avalanche Research SLF, Davos Dorf, Switzerland
[8]Wyss Academy for Nature, University of Bern, Bern, Switzerland
[9]Climate and Environmental Physics, Physics Institute, University of Bern, Bern, Switzerland
[10]Oeschger Centre for Climate Change Research, University of Bern, Bern, Switzerland
[11]Center for Climate Systems Modeling, ETH Zurich, Zurich, Switzerland
[12]Institute of Biology/Geobotany and Botanical Garden, Martin Luther University Halle-Wittenberg, Halle, Germany
[13]German Centre for Integrative Biodiversity Research (iDiv) Halle-Jena-Leipzig, Leipzig, Germany
[14]Senckenberg Biodiversity and Climate Research Centre, Frankfurt am Main, Germany
[15]Department of Physical Geography, Goethe University, Frankfurt am Main, Germany
[16]Faculty of Geotechnical Engineering, University of Zagreb, Varaždin, Croatia
[17]Department of Plant Biology and Ecology, University of the Basque Country UPV/EHU, Bilbao, Spain
[18]Biology Education, Dokuz Eylul University, Izmir, Turkey
[19]Vegetation Ecology Research Group, Institute of Natural Resource Management (IUNR), Zurich University of Applied Sciences (ZHAW), Wädenswil, Switzerland
[20]Bayreuth Center of Ecology and Environmental Research (BayCEER), University of Bayreuth, Bayreuth, Germany
[21]Institute for Environmental Studies, Vrije Universiteit Amsterdam, Amsterdam, Netherlands
*Corresponding author (petra.sieber@env.ethz.ch)

Contents

Supplementary Note 1: Modelling and mapping the distribution of EUNIS level 3 habitats

Supplementary Note 2: Disaggregation of EUNIS level 3 habitat types into plant functional types

Supplementary Note 3: Comparison of MPI-ESM1-2-HR with other CMIP6 models over Europe

Supplementary Note 4: Evaluation of the COSMO-CLM^2 regional Earth System Model

Supplementary Figures

Supplementary Tables

Supplementary References

Supplementary Note 1: Modelling and mapping the distribution of EUNIS level 3 habitats

We build on a recent effort[1] to model thematic habitats at 1 km resolution across Europe, following the four land system maps of Dou et al.[2] and climate under RCP2.6 (Supplementary Fig. 16). The EUNIS habitat classification[3] provides a hierarchical system to classify European habitats into broad groups (level 1) and more fine-grained habitat types (levels 2 and 3). This work considers 260 different habitat types at level 3 belonging to eight groups at level 1: saltmarshes, coastal habitats, fresh water, wetlands, grasslands, shrublands, forests, and unvegetated or sparsely vegetated habitats.

We build a set of ensemble models to classify EUNIS habitat types at level 3 within each group (level 1), given environmental conditions including climate, terrain, geology, hydrography and soil properties (Supplementary Table 2). A multi-class objective is used to produce a probabilistic ranking of habitat types. The ensemble models comprise state-of-art machine learning algorithms from three families: bagging (random forest), boosting (XGBoost, lightGBM, CatBoost), and neural networks (multi-layer perceptrons and tabular attention networks). Each algorithm's hyperparameters are automatically tuned using Bayesian parameter search through the *optuna* package, and we keep the best performing models (balanced accuracy >0.3) of each family. For calibration we use plots from the European Vegetation Archive (EVA)[4] classified as EUNIS level 3 habitat types according to the classification expert system EUNIS-ESy[5,6]. The models are trained and evaluated in a stratified spatial block cross-validation. Validation is performed with independent regional maps from Austria, France, the Netherlands, and Portugal.

For each land system map, we use crosswalk rules (Supplementary Data 1) to convert the land system in each pixel to a EUNIS level 1 group. Then, we use the ensemble model of the selected group to predict the most likely EUNIS level 3 habitat type (Supplementary Table 3). In mosaic land systems, several EUNIS level 1 groups can be selected following the previous rule. For example, agricultural mosaics are a combination of pastures (grasslands) and croplands (man-made vegetation). To resolve this, we additionally consider a ranking of EUNIS habitat groups based on their relative frequency in each mosaic land system.

Supplementary Note 2: Disaggregation of EUNIS level 3 habitat types into plant functional types

The habitat types at EUNIS level 3 are disaggregated into plant functional types (PFTs) of CLM5 (Supplementary Fig. 17) according to the average species cover in selected vegetation plots (see Supplementary Figs. 18-19 and Supplementary Table 4 for vegetation plots by habitat type and database). For this purpose, we obtained 1,263,388 georeferenced vegetation plots from EVA[4] (EVA project 123, data retrieved on 5 May 2021). We selected plots that were classified into EUNIS level 3 habitat types according to the expert system EUNIS-ESy[5,6], sized 1-1000 m^2, and below coordinate uncertainty of 5 km. Plots with unknown plot size and coordinate uncertainty were retained to ensure sufficient geographical coverage (e.g., most of France would have been lost otherwise). This selection resulted in the 819,232 plots used in the study. After plant species to PFTs, we compute the average cover of individual PFTs across plots for 235 habitat types (Supplementary Data 2).

In the vegetation plot data of EVA, species cover is provided as percent cover of the surface after transformation from cover class scales such as Braun-Blanquet. For instance, a dense forest with a dense understory herb-layer is considered to have 100% tree cover and nearly 100% herb cover. In contrast, in the land surface model CLM5 fractions of (semi-)natural vegetation PFTs must sum to 100% and only the highest vegetation layer is considered for flux calculations (i.e., considering a vertical structure of trees, shrubs, herb-layer plants, and bare soil)[7]. To achieve this, the PFT cover values derived from EVA plot data need to be adjusted.

First, we classify natural vegetation PFTs into three layers L = (trees, shrubs, herb-layer) depending on their position in the vertical vegetation structure. The categories are composed of PFTs as follows: trees = (Needleleaf evergreen tree – temperate, Needleleaf evergreen tree – boreal, Needleleaf deciduous tree – boreal, Broadleaf evergreen tree – tropical, Broadleaf evergreen tree – temperate, Broadleaf deciduous tree – tropical, Broadleaf deciduous tree – temperate, Broadleaf deciduous tree – boreal), shrubs = (Broadleaf evergreen shrub – temperate, Broadleaf deciduous shrub – temperate, Broadleaf deciduous shrub – boreal), herb-layer = (C_3 arctic grass, C_3 grass, C_4 grass). For each layer, the total cover is calculated by summing over the PFTs within that layer.

Second, the cover of each layer (C_L) is adjusted depending on its position in the vertical vegetation structure:

If $C_{\text{tree}} \geq 100$:

$C^*_{\text{tree}} = 100$
$C^*_{\text{shrub}} = 0$
$C^*_{\text{herb}} = 0$

If $C_{\text{tree}} < 100$:

$C^*_{\text{tree}} = C_{tree}$
$C^*_{\text{shrub}} = C_{\text{shrub}} - C_{\text{tree}}$

If $C^*_{\text{tree}} + C^*_{\text{shrub}} \geq 100$:

$C^*_{\text{shrub}} = 100 - C^*_{tree}$
$C^*_{\text{herb}} = 0$

If $C^*_{\text{tree}} + C^*_{\text{shrub}} < 100$:

If $C^*_{\text{tree}} + C^*_{\text{shrub}} + C^*_{\text{herb}} \geq 100$:

$C^*_{\text{herb}} = 100 - C^*_{\text{tree}} - C^*_{\text{shrub}}$

Third, the total vegetation cover C_{veg} is calculated as the sum of the adjusted cover values C^*_{tree}, C^*_{shrub}, and C^*_{herb}. If the resulting C_{veg} is <100, bare soil covers the rest:

$C_{\text{bare}} = 100 - C_{\text{veg}}$

Finally, the PFT fractions within each layer are scaled proportionally so that they sum to the adjusted cover value.

Comparison with existing PFT and land cover maps

We apply the EUNIS habitat mapping and translation into PFTs to a land systems map for the year 2015[2] and compare the results with the original CLM5 land surface characterisation based on satellite-observed land cover in 2005 (Supplementary Table 5). Furthermore, we compare both with CORINE Land Cover data for 2018[8]. Compared with CLM5, our method yields greater cover of bare soil, shrubs, crops, and broadleaf trees, and lower cover of grass PFTs and needleleaf trees. The fractions of bare soil and shrubs, and the greater proportion of croplands than grasslands agree better with CORINE Land Cover in our results than in the original CLM5 dataset. The lower cover of needleleaf trees is partly due to differences in the definition of tree cover vs. forest cover (e.g., forests are considered as formations with at least 60% canopy cover by the satellite-based MODIS Land Cover product used for CLM5, and 30% canopy cover by CORINE Land Cover). However, we cannot rule out that our method underestimates tree cover, particularly the optimal tree cover for flux calculations in CLM5, which is calibrated based on satellite-observed land cover and leaf area index (LAI). While additional evaluation and model benchmarking is needed, our methods considering spatially explicit habitat and species information could be a viable alternative to spatially uniform crosswalk rules used to disaggregate land cover classes into PFTs[9,10].

Supplementary Note 3: Comparison of MPI-ESM1-2-HR with other CMIP6 models over Europe

For the future scenarios, COSMO-CLM[2] is driven with boundary conditions derived from the global climate model MPI-ESM1-2-HR under SSP1-2.6, using the first realisation (r1i1p1f1)[11]. MPI-ESM1-2-HR is chosen due to good performance over Europe[12], medium equilibrium climate sensitivity, and high spatial resolution (~100 km for land and atmosphere). For these reasons, MPI-ESM1-2-HR is also used for CORDEX-CMIP6 simulations over Europe[13] and the CORDEX Flagship Pilot Study LUCAS, which focuses specifically on the response to land use change forcing.

To understand how MPI-ESM1-2-HR compares to other CMIP6 models used to drive regional climate simulations over Europe, we evaluate six global climate models (GCMs) selected for the EURO-CORDEX CMIP6 balanced matrix experiment[13]. Among EURO-CORDEX experiments, this experiment includes the largest number of GCMs and aims to cover a wide range of possible climate outcomes and uncertainties at acceptable computational cost. The included models are CMCC-CM2-SR5, CNRM-ESM2-1, EC-Earth3-Veg, MIROC6, MPI-ESM1-2-HR, and NorESM2-MM and we use the first available realisation for the SSP1-2.6 scenario. A common way to compare potential driving GCMs is to characterise them in terms of spatial and temporal means of near-surface air temperature and precipitation[12], which are closely linked to the boundary fields used to drive regional climate models.

Our evaluation shows that MPI-ESM1-2-HR falls into the range of annual and seasonal mean values of temperature and precipitation simulated by the other GCMs over Europe in 2036-2050 (Supplementary Fig. 21). Only MIROC6 and CMCC-CM2-SR5 consistently project warmer annual and summer mean temperatures in all subregions. Compared with the other GCMs, MPI-ESM1-2-HR is usually neither the coldest, warmest, driest, or wettest model. The six GCMs together cover a wide range of annual (~5 °C) and summer (~6 °C) mean temperatures across models and years, whereas a single GCM (here focusing on MPI-ESM1-2-HR) covers a range of ~1.5 °C across years (Supplementary Fig. 21). Interannual variation in MPI-ESM1-2-HR amounts to ~31% (24-48% in subregions) of multi-model interannual variation for annual mean temperature, and 56% (45-79% in subregions) for annual mean precipitation, while temperature and precipitation per model are weakly correlated. Interannual variation in temperature and precipitation of MPI-ESM1-2-HR is thus useful to test the robustness of the climate response under varying background climate, but it does not sample the full range of possible climate conditions.

Supplementary Note 4: Evaluation of the COSMO-CLM[2] regional Earth System Model

COSMO-CLM[2] is evaluated against the station-based gridded product E-OBS[14] version 28.0e at 0.1° horizontal resolution, using seasonal means (2011-2015) of 2 m air temperature and precipitation (Supplementary Fig. 20). For this purpose, we restrict the E-OBS dataset to grid cells that lie within 100 km of a station that was active in 2011-2015 to remove data-scarce areas. The mean absolute error (MAE) in different seasons is within 0.61-0.82 °C for temperature and 0.38-0.55 mm day^{-1} for precipitation (Supplementary Table 6). This bias is only slightly greater than that of ERA5-Land[15] compared with E-OBS (0.47-0.66 °C for temperature and 0.42-0.49 mm day^{-1} for precipitation), indicating uncertainties in the ERA5 boundary conditions for COSMO-CLM[2] and/or the E-OBS dataset as an observational reference. The performance of COSMO-CLM[2] consisting of COSMO-Model 6 and CLM5 driven with ERA5 is similar to the previous version consisting of COSMO-Model 6 and CLM4.5 driven with ERAInterim, with notable improvements in summer-time temperatures.

Supplementary Figures

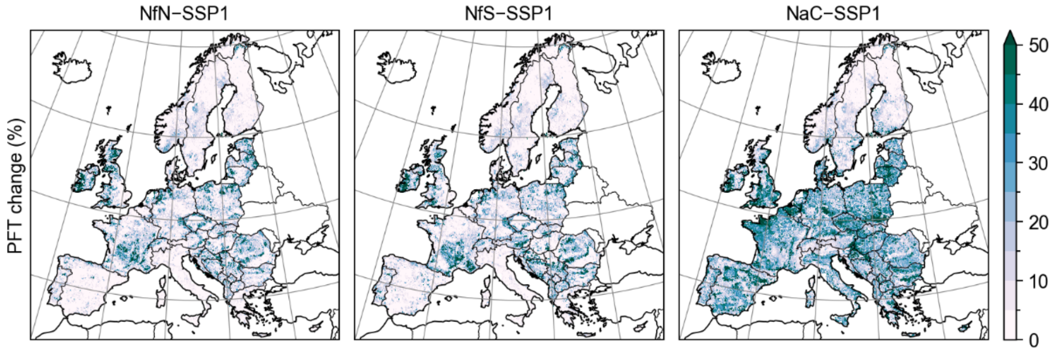

Supplementary Fig. 1. Level of perturbation. Grid cell fraction affected by changes in plant functional types (PFTs) in the scenarios Nature for Nature (NfN), Nature for Society (NfS), and Nature as Culture (NaC) relative to the reference (SSP). Changes are calculated as net transitions at the grid cell level and then aggregated, such that opposing transitions cancel within grid cells but not between grid cells.

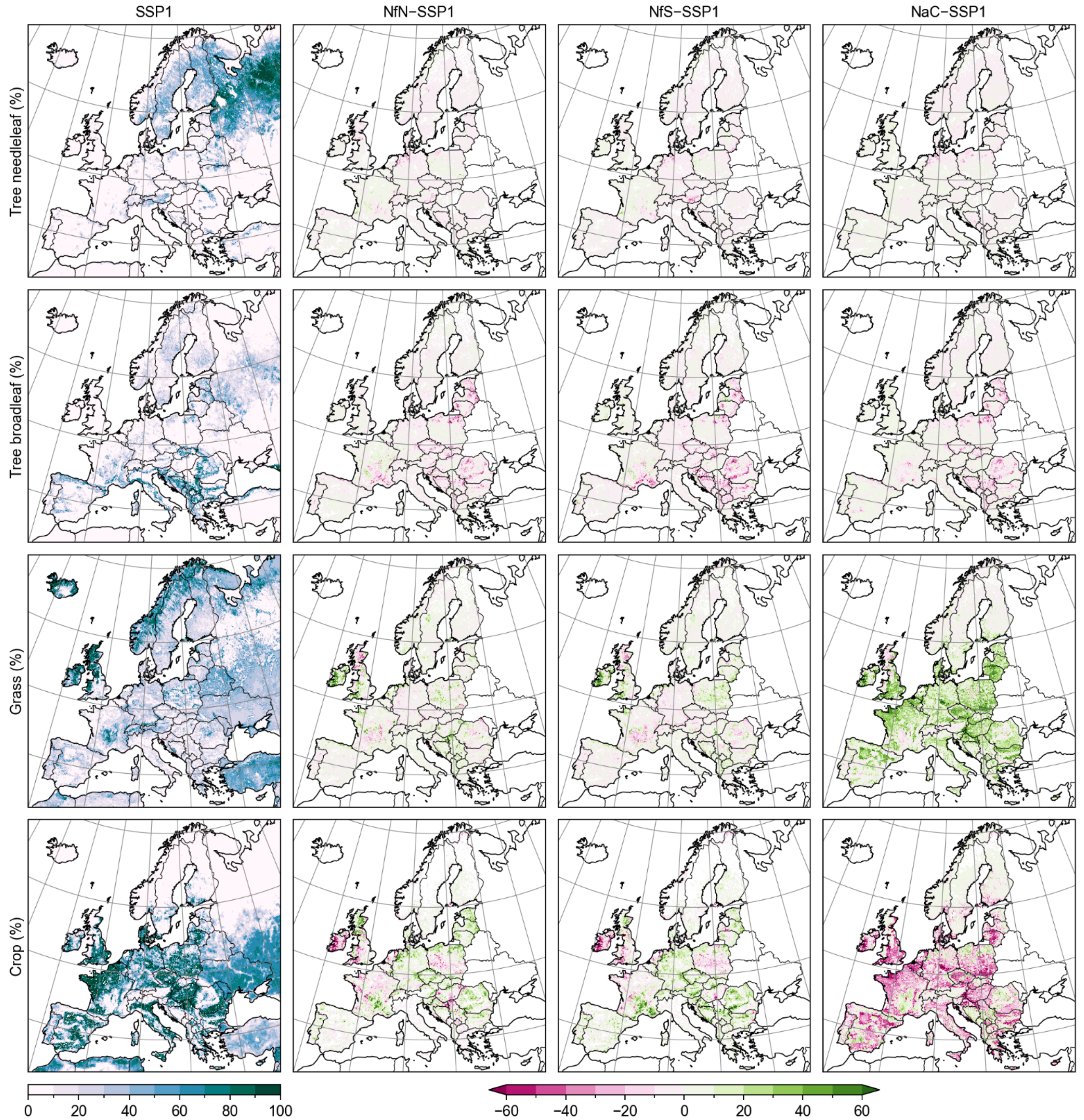

Supplementary Fig. 2. Land cover based on fractions of plant functional types (PFTs). Absolute values in the reference (SSP1, left) and changes in the scenarios Nature for Nature (NfN), Nature for Society (NfS), and Nature as Culture (NaC) relative to SSP1 (right). PFT variants such as temperate and boreal types, deciduous and evergreen trees, C3 and C4 grasses, or rainfed and irrigated crops are summed. Shrubs and bare soil are not shown.

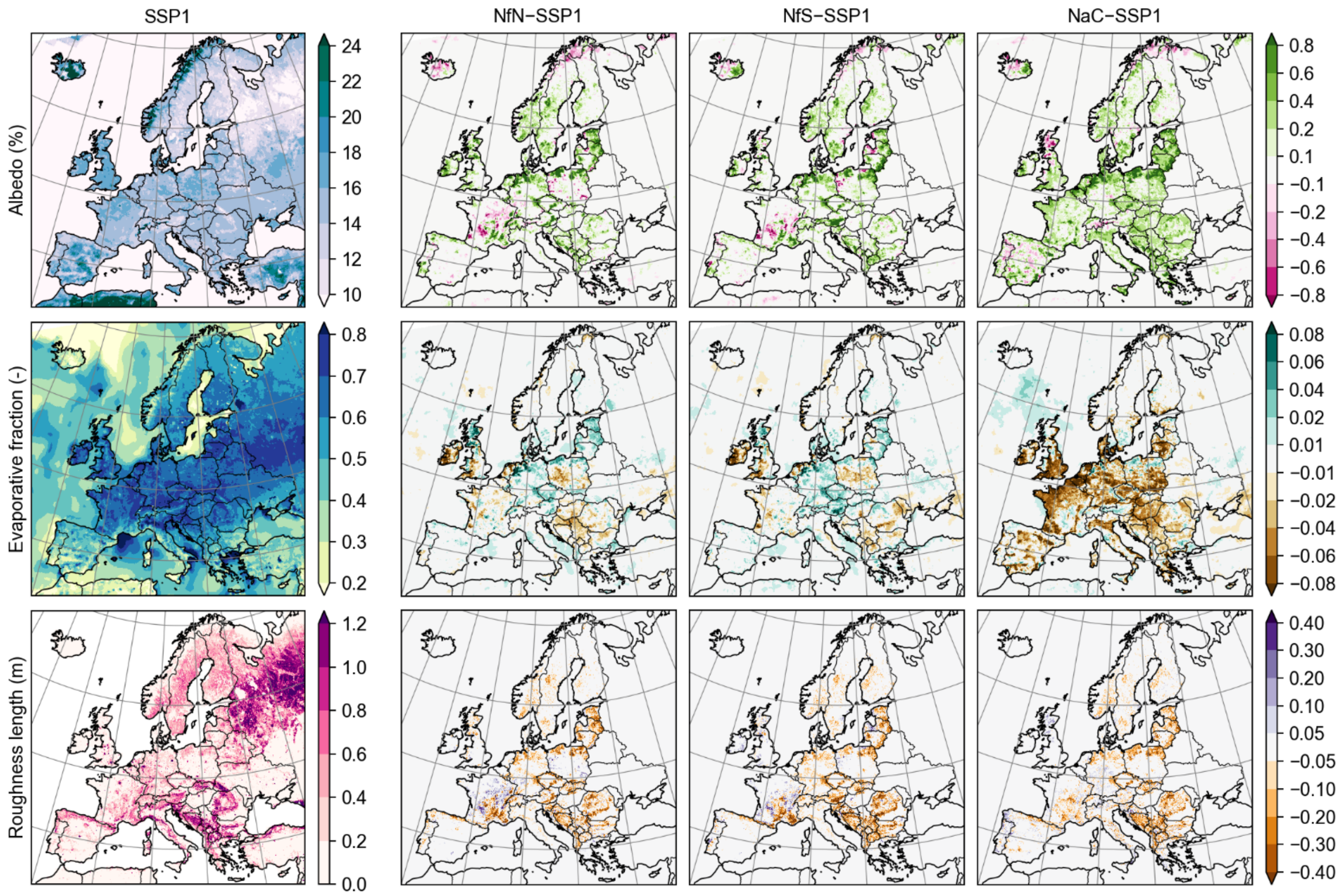

Supplementary Fig. 3. Emergent biogeophysical surface characteristics. Summer (JJA) climatology (2036-2050) of biogeophysical variables in the reference (SSP1) and changes in the scenarios Nature for Nature (NfN), Nature for Society (NfS), and Nature as Culture (NaC) relative to SSP1.

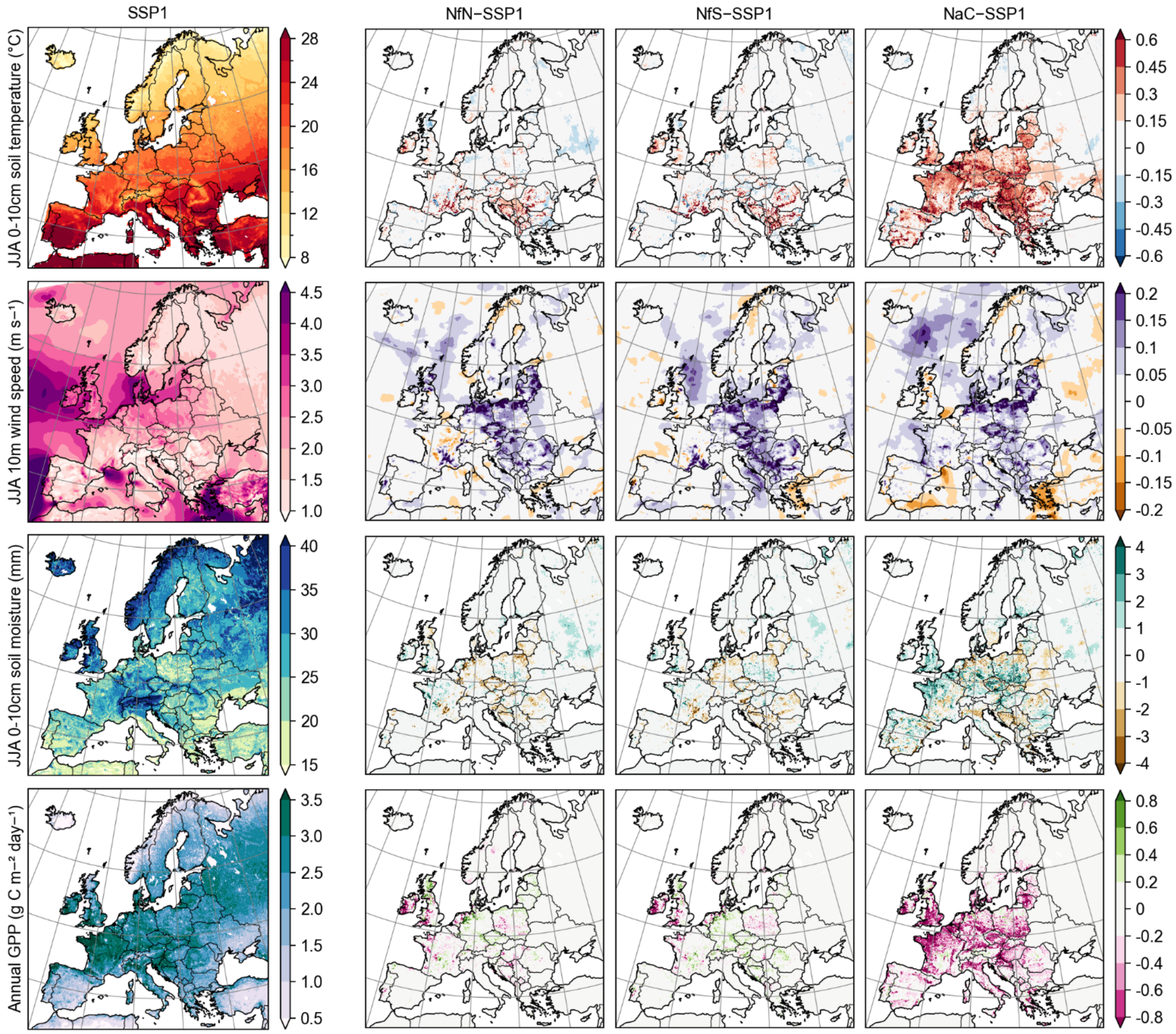

Supplementary Fig. 4. Climate response to Nature Future scenarios. Climate (2036-2050 mean) in the reference (SSP1, left) and responses in the scenarios Nature for Nature (NfN), Nature for Society (NfS), and Nature as Culture (NaC) relative to SSP1 (right). Summer (JJA) soil temperature at 0-10 cm, summer wind speed at 10 m, summer soil moisture at 0-10 cm, and annual gross primary production (GPP).

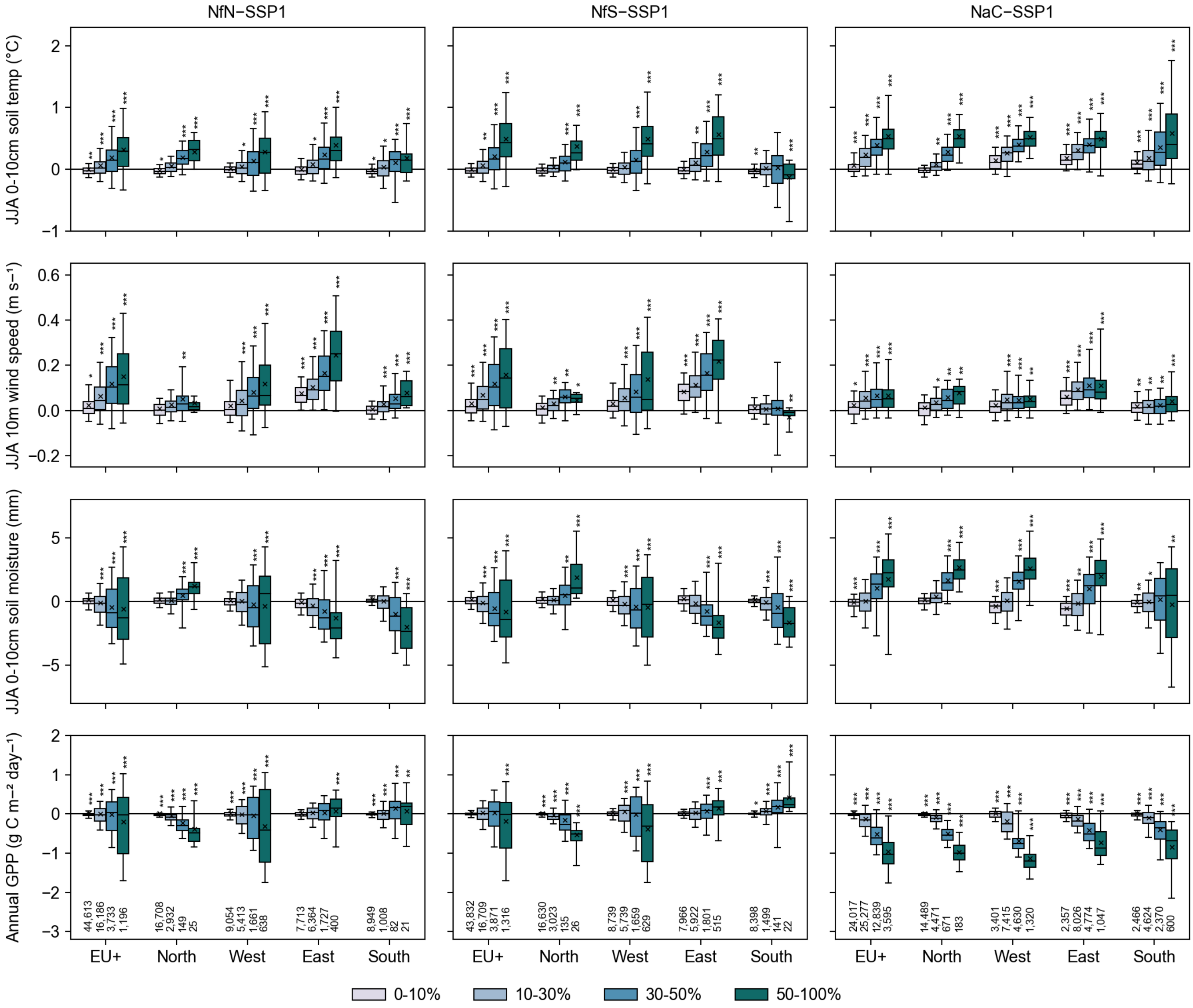

Supplementary Fig. 5. Climate sensitivity to changes in plant functional types over Europe (EU+) and in subregions. For the scenarios Nature for Nature (NfN), Nature for Society (NfS), and Nature as Culture (NaC) relative to the reference (SSP1), responses in the 2036-2050 mean summer (JJA) soil temperature at 0-10 cm, summer wind speed at 10 m, summer soil moisture at 0-10 cm, and annual gross primary production (GPP) at the grid cell level are grouped by the proportion of changes in plant functional types (0-10%, 10-30%, 30-50%, or 50-100% of the grid cell). Numbers in the bottom row indicate the number of grid cells included per group to generate the boxplot. Boxes indicate the first quantile, the third quantile, and the median of grid cell level data, and whiskers extend from the 5th to the 95th percentile. Crosses indicate the area-weighted mean. Statistical significance is based on two-sided Wilcoxon signed-rank tests, with p-values adjusted for multiple comparisons across groups and regions (*P < 0.1, **P < 0.05, ***P < 0.01).

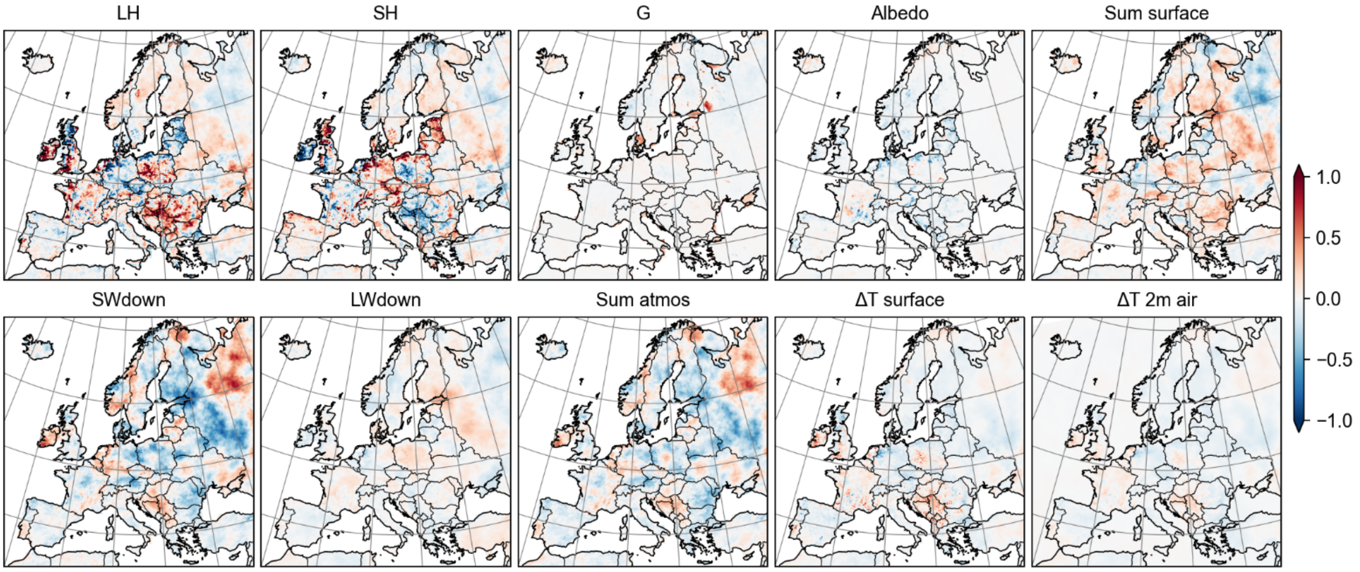

Supplementary Fig. 6. Contributions of surface energy balance components to the grid cell level surface temperature response in summer (2036-2050 mean) in the Nature for Nature (NfN) scenario. Contributions at the land surface include latent heat flux (LH), sensible heat flux (SH), ground heat flux (G), and albedo, whereas those of atmospheric feedbacks are given by downwelling shortwave radiation (SWdown) and downwelling longwave radiation (LWdown). The simulated responses in surface (skin) temperature and near-surface (2 m) air temperature are shown for comparison.

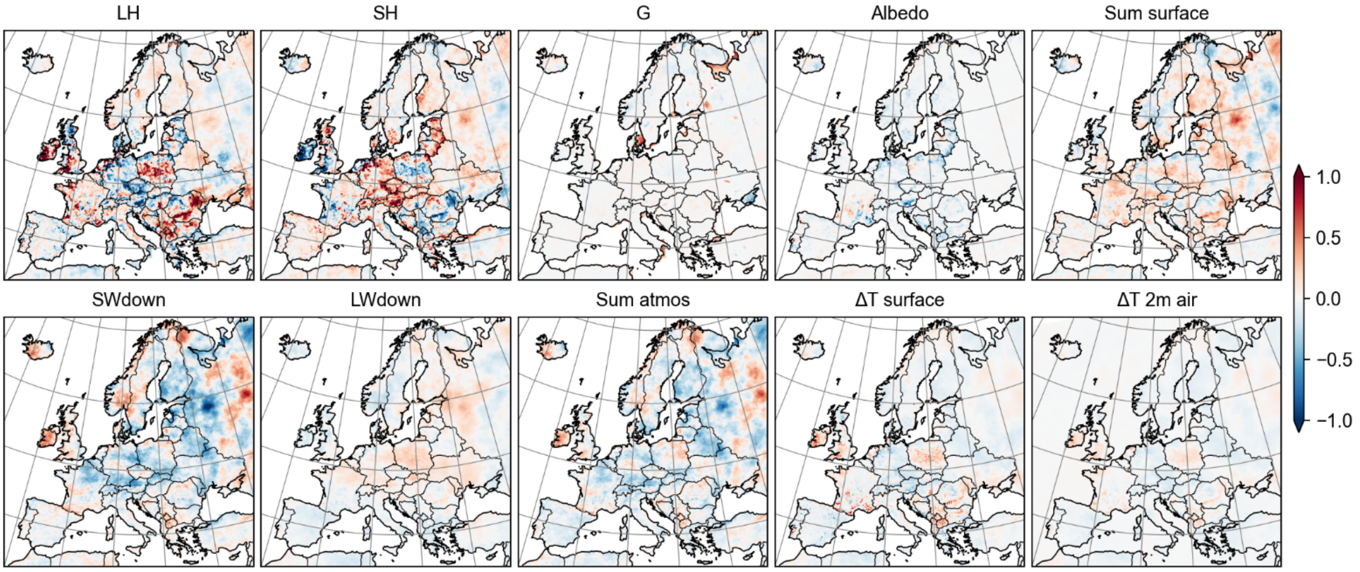

Supplementary Fig. 7. Contributions of surface energy balance components to the grid cell level surface temperature response in summer (2036-2050 mean) in the Nature for Society (NfS) scenario. Contributions at the land surface include latent heat flux (LH), sensible heat flux (SH), ground heat flux (G), and albedo, whereas those of atmospheric feedbacks are given by downwelling shortwave radiation (SWdown) and downwelling longwave radiation (LWdown). The simulated responses in surface (skin) temperature and near-surface (2 m) air temperature are shown for comparison.

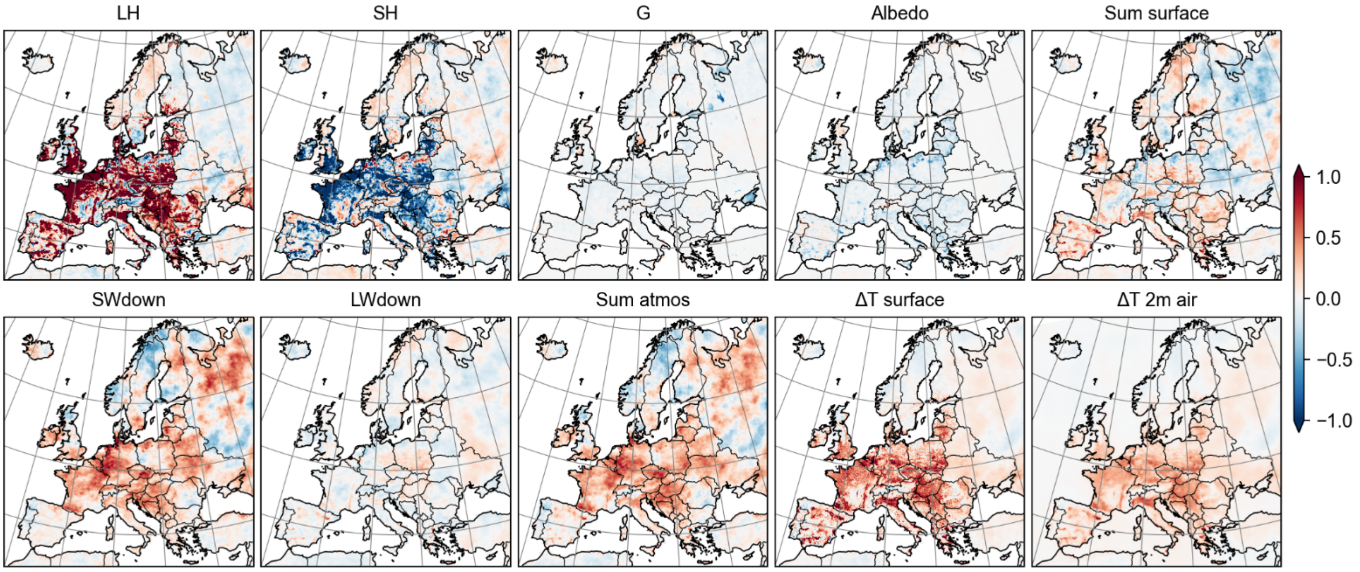

Supplementary Fig. 8. Contributions of surface energy balance components to the grid cell level surface temperature response in summer (2036-2050 mean) in the Nature as Culture (NaC) scenario. Contributions at the land surface include latent heat flux (LH), sensible heat flux (SH), ground heat flux (G), and albedo, whereas those of atmospheric feedbacks are given by downwelling shortwave radiation (SWdown) and downwelling longwave radiation (LWdown). The simulated responses in surface (skin) temperature and near-surface (2 m) air temperature are shown for comparison.

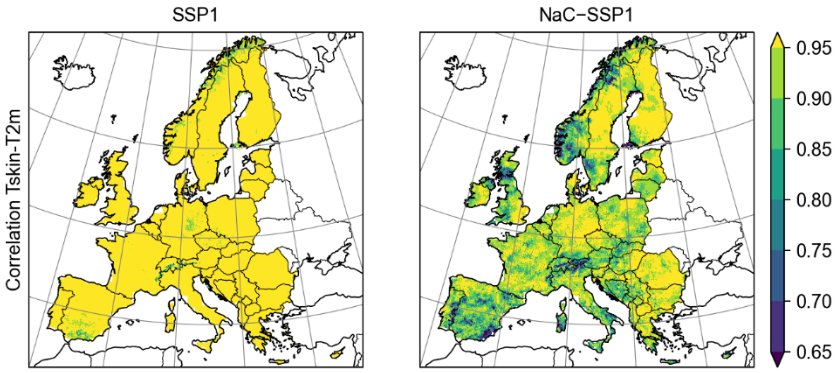

Supplementary Fig. 9. Correlation of surface skin temperature and near-surface (2 m) air temperature. Absolute temperatures in the reference (SSP1, left) and temperature response in the Nature as Culture (NaC) scenario relative to SSP1 (right).

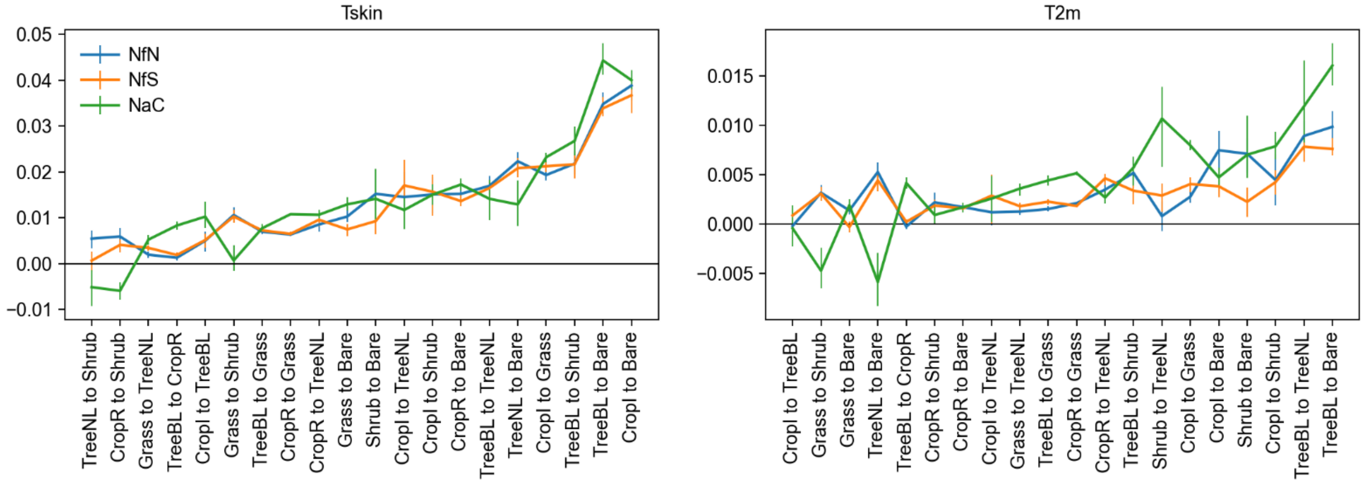

Supplementary Fig. 10. Temperature sensitivity to individual land cover transitions over Europe. Regressions are performed on the 2036-2050 mean temperature response. Sensitivities are given by the unstandardised regression coefficients for surface skin temperature (left) and near-surface (2 m) air temperature (right), estimated for the scenarios Nature for Nature (NfN), Nature for Society (NfS), and Nature as Culture (NaC). Land cover transitions are named by the dominant direction across scenarios and sorted by average strength across scenarios. Thick lines show the mean across 200 linear regression fits and error bars extend from the 2.5th to the 97.5th percentile of the bootstrap sample.

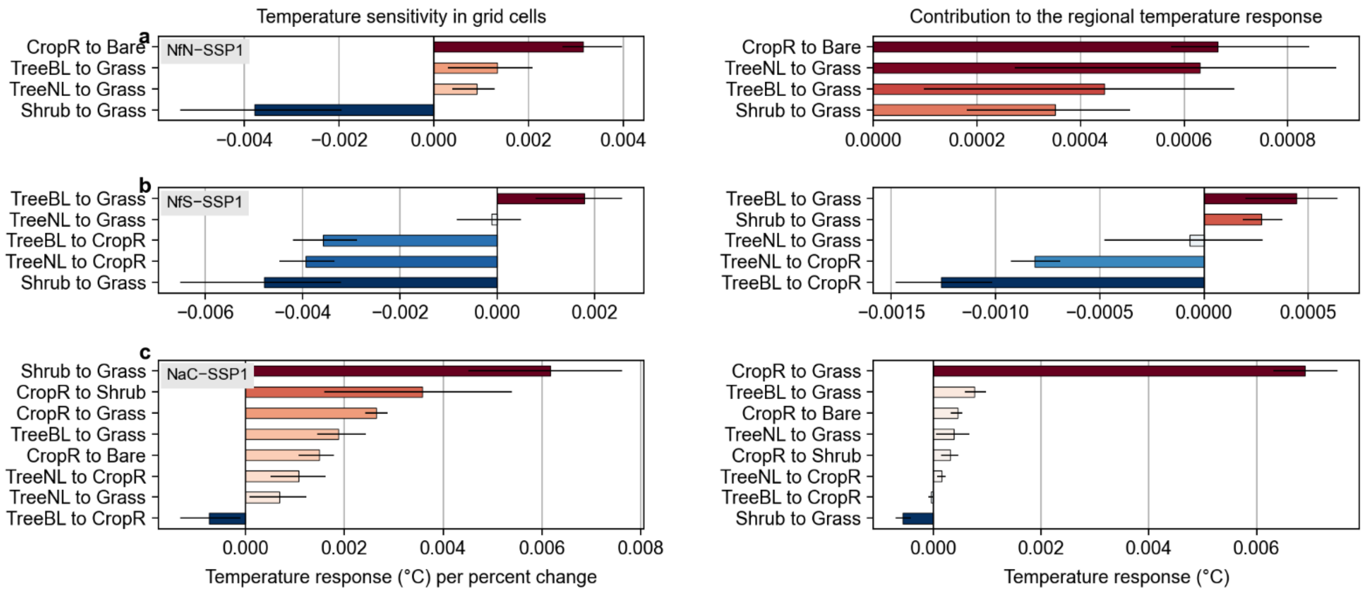

Supplementary Fig. 11. Contribution of individual land cover transitions to the 2 m air temperature response in summer over the North subregion. Regressions are performed on the 2036-2050 mean temperature response. Sensitivities are given by the unstandardised regression coefficients and contributions by the sensitivities multiplied by the percentage change over the region. Results are shown for **a**, the Nature for Nature (NfN) scenario, **b**, the Nature for Society (NfS) scenario, and **c**, the Nature as Culture (NaC) scenario. For each scenario, land cover transitions are shown that occur on >0.2% of the area and in >20% of grid cells. Land cover transitions are named by the dominant direction per scenario and sorted by strength of effect. Bars show the mean across 200 linear regression fits and error bars extend from the 2.5th to the 97.5th percentile of the bootstrap sample.

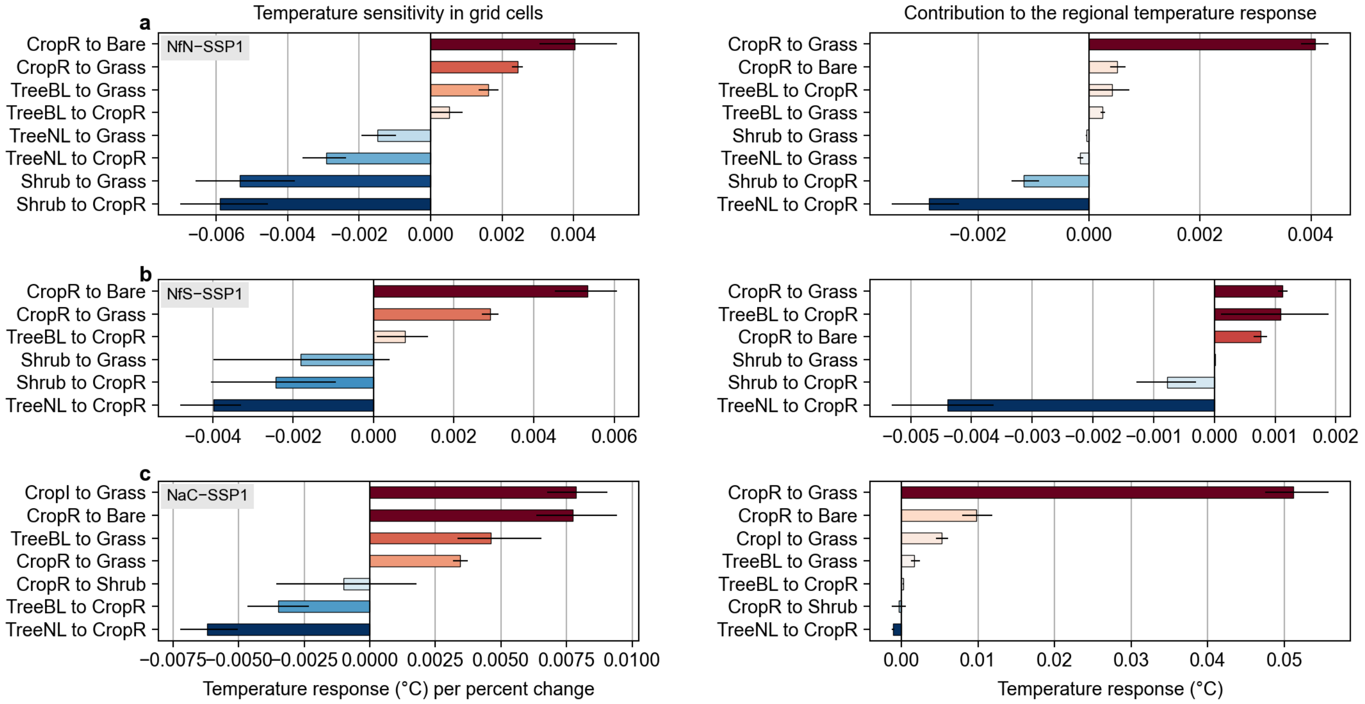

Supplementary Fig. 12. Contribution of individual land cover transitions to the 2 m air temperature response in summer over the West subregion. Regressions are performed on the 2036-2050 mean temperature response. Sensitivities are given by the unstandardised regression coefficients and contributions by the sensitivities multiplied by the percentage change over the region. Results are shown for **a**, the Nature for Nature (NfN) scenario, **b**, the Nature for Society (NfS) scenario, and **c**, the Nature as Culture (NaC) scenario. For each scenario, land cover transitions are shown that occur on >0.2% of the area and in >20% of grid cells. Land cover transitions are named by the dominant direction per scenario and sorted by strength of effect. Bars show the mean across 200 linear regression fits and error bars extend from the 2.5th to the 97.5th percentile of the bootstrap sample.

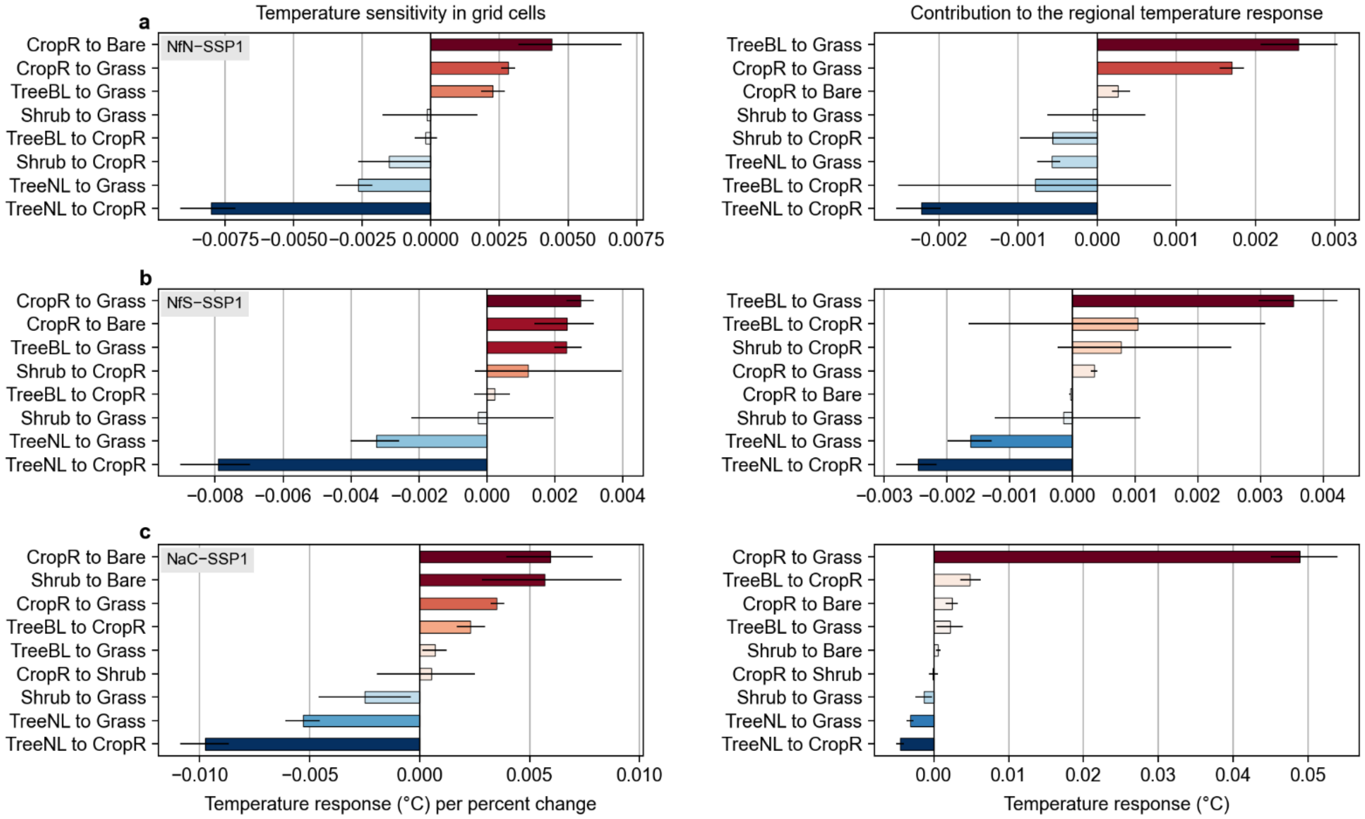

Supplementary Fig. 13. Contribution of individual land cover transitions to the 2 m air temperature response in summer over the East subregion. Regressions are performed on the 2036-2050 mean temperature response. Sensitivities are given by the unstandardised regression coefficients and contributions by the sensitivities multiplied by the percentage change over the region. Results are shown for **a**, the Nature for Nature (NfN) scenario, **b**, the Nature for Society (NfS) scenario, and **c**, the Nature as Culture (NaC) scenario. For each scenario, land cover transitions are shown that occur on >0.2% of the area and in >20% of grid cells. Land cover transitions are named by the dominant direction per scenario and sorted by strength of effect. Bars show the mean across 200 linear regression fits and error bars extend from the 2.5th to the 97.5th percentile of the bootstrap sample.

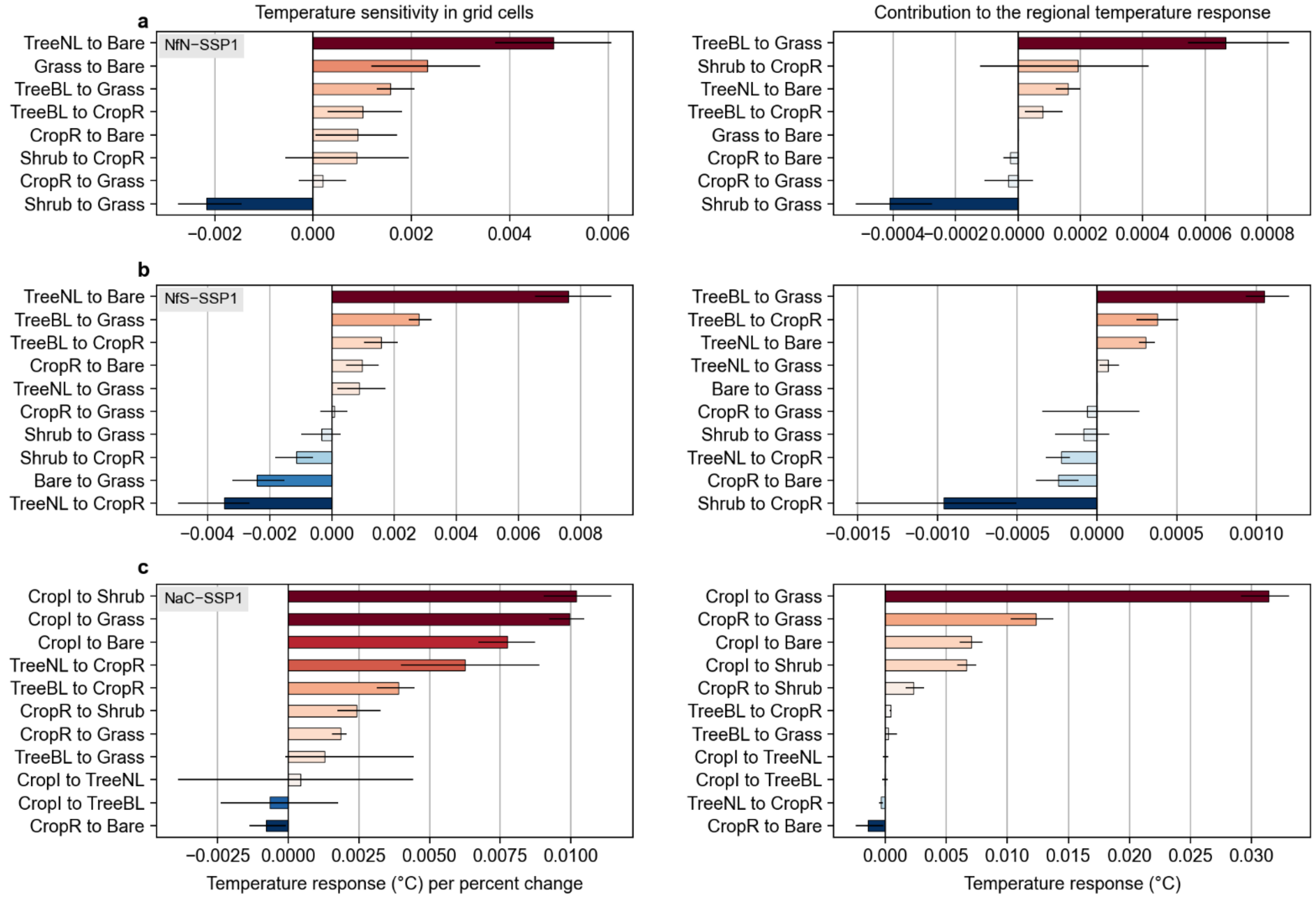

Supplementary Fig. 14. Contribution of individual land cover transitions to the 2 m air temperature response in summer over the South subregion. Regressions are performed on the 2036-2050 mean temperature response. Sensitivities are given by the unstandardised regression coefficients and contributions by the sensitivities multiplied by the percentage change over the region. Results are shown for **a**, the Nature for Nature (NfN) scenario, **b**, the Nature for Society (NfS) scenario, and **c**, the Nature as Culture (NaC) scenario. For each scenario, land cover transitions are shown that occur on >0.2% of the area and in >20% of grid cells. Land cover transitions are named by the dominant direction per scenario and sorted by strength of effect. Bars show the mean across 200 linear regression fits and error bars extend from the 2.5th to the 97.5th percentile of the bootstrap sample.

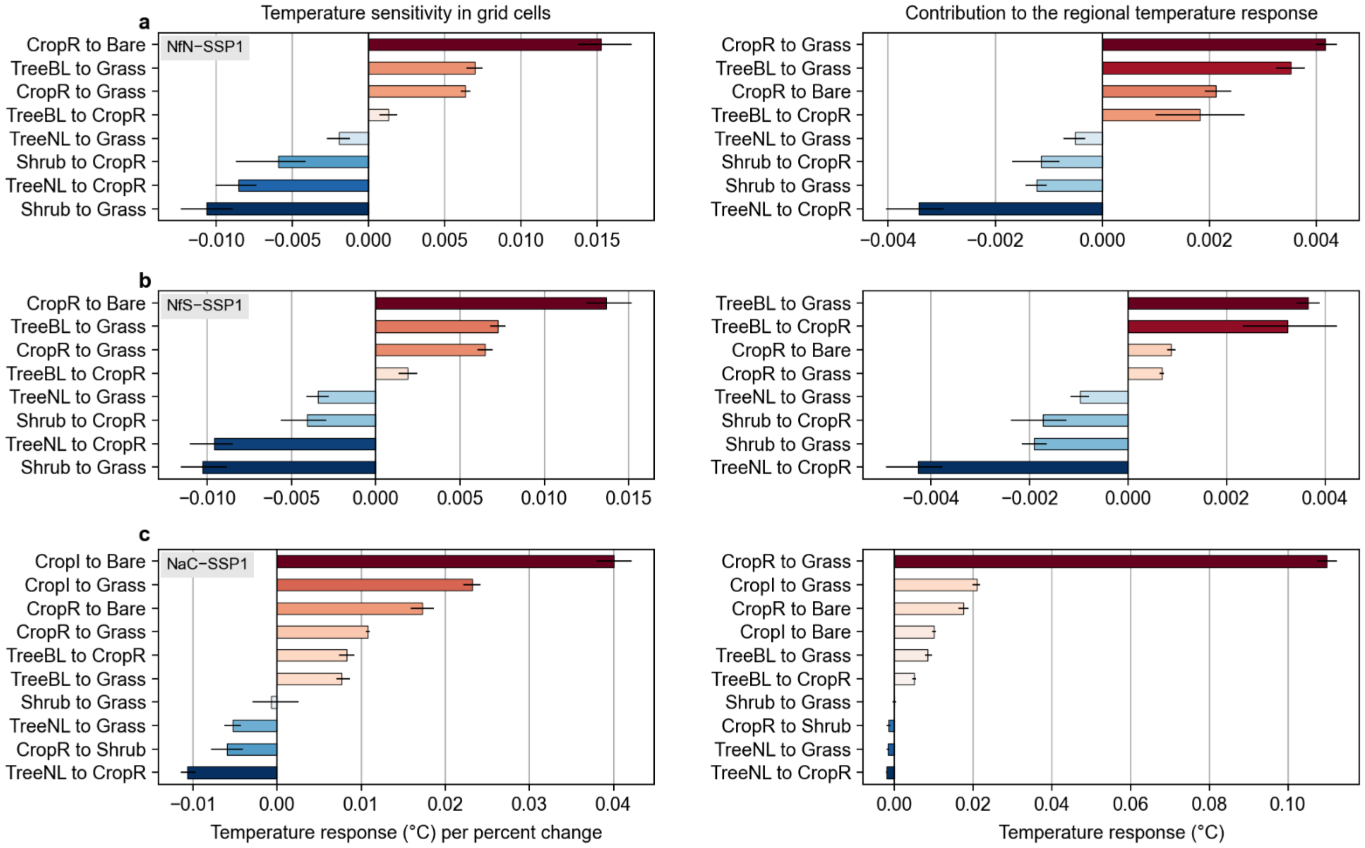

Supplementary Fig. 15. Contribution of individual land cover transitions to the surface skin temperature response in summer over Europe. Regressions are performed on the 2036-2050 mean temperature response. Sensitivities are given by the unstandardised regression coefficients and contributions by the sensitivities multiplied by the percentage change over the region. Results are shown for **a**, the Nature for Nature (NfN) scenario, **b**, the Nature for Society (NfS) scenario, and **c**, the Nature as Culture (NaC) scenario. For each scenario, land cover transitions are shown that occur on >0.2% of the area and in >20% of grid cells. Land cover transitions are named by the dominant direction per scenario and sorted by strength of effect. Bars show the mean across 200 linear regression fits and error bars extend from the 2.5th to the 97.5th percentile of the bootstrap sample.

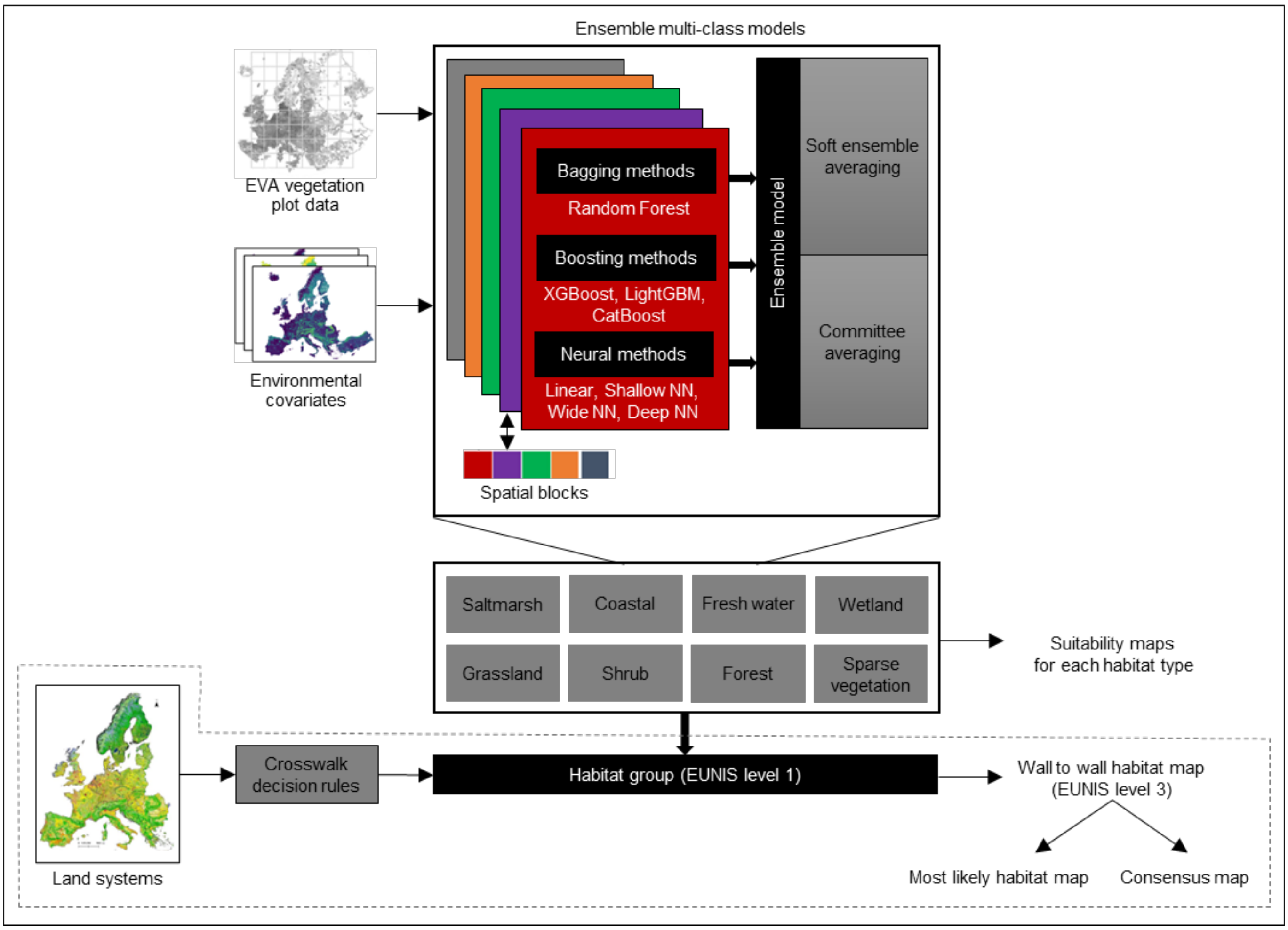

Supplementary Fig. 16. Workflow for modelling and mapping the distribution of habitat types. Ensemble multi-class models are trained for each European Nature Information System (EUNIS) level 1 habitat group, using vegetation plot data from the European Vegetation Archive (EVA) and environmental covariates. Each land system is linked to a habitat group at EUNIS level 1 through crosswalk decision rules, and the corresponding ensemble model is used to predict the most likely habitat type at EUNIS level 3.

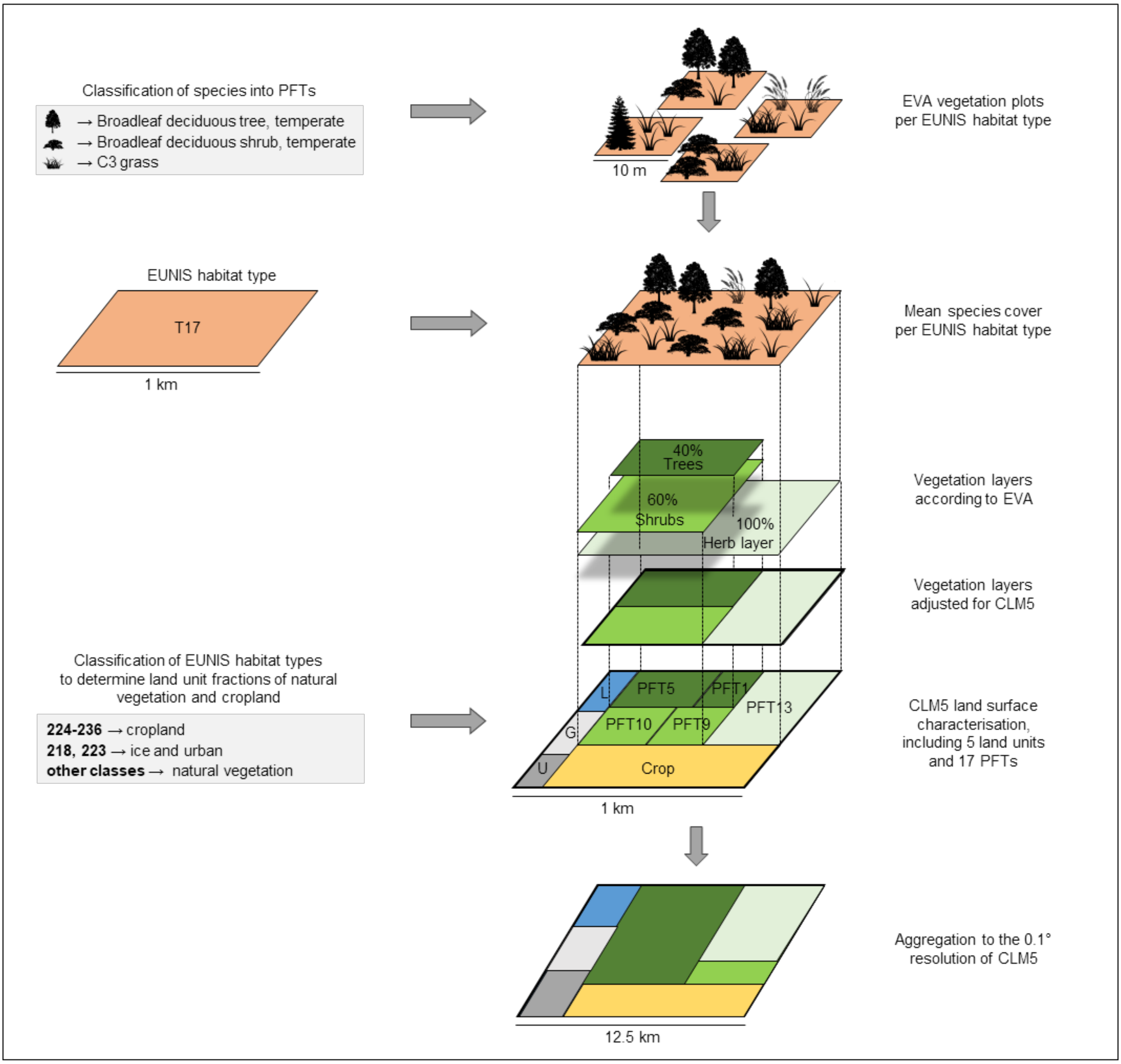

Supplementary Fig. 17. **Workflow for the modification of CLM5 plant functional type (PFT) fractions based on species cover in habitat types.** For each European Nature Information System (EUNIS) habitat type at level 3, the average species cover is calculated from vegetation plots of the European Vegetation Archive (EVA). Each species is assigned to a PFT and the total cover per PFT is calculated. PFTs are classified into vegetation layers (trees, shrubs, or herb-layer) and the PFT cover is adjusted to account for the vertical vegetation structure in CLM5. The relative cover of natural vegetation and crops is modified directly based on EUNIS classes classified into land cover types. The data is aggregated to the 0.1° grid used in CLM5 and used to replace the PFT fractions on the natural vegetation land unit and to modify the proportions of natural vegetation and cropland. The other land units (L = lake, G = glacier, U = urban) are not modified.

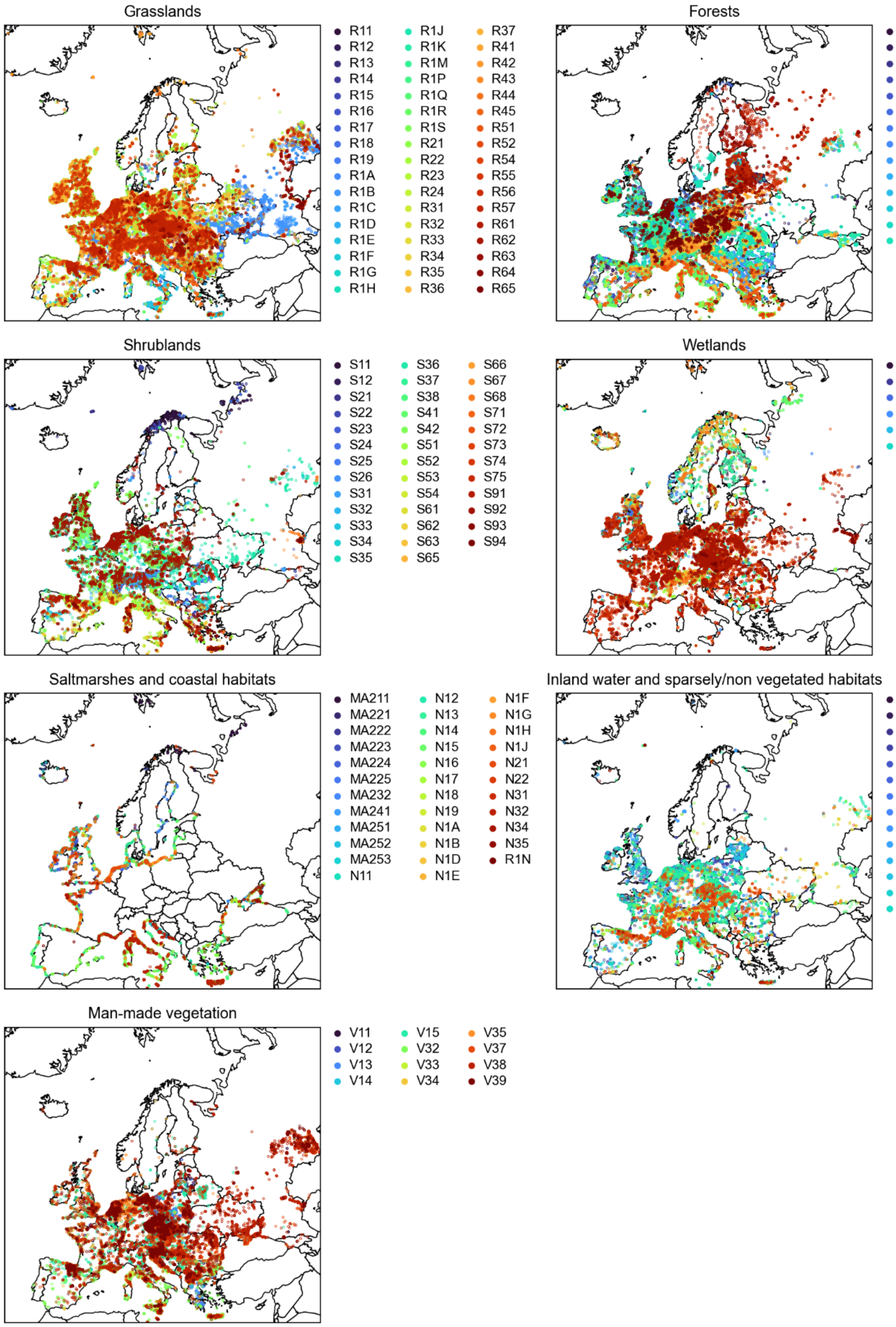

Supplementary Fig. 18. Vegetation plots used in this study, split into habitat groups and colored by EUNIS level 3 habitat type. The dataset includes 819,232 individual plots of the European Vegetation Archive. EUNIS = European Nature Information System.

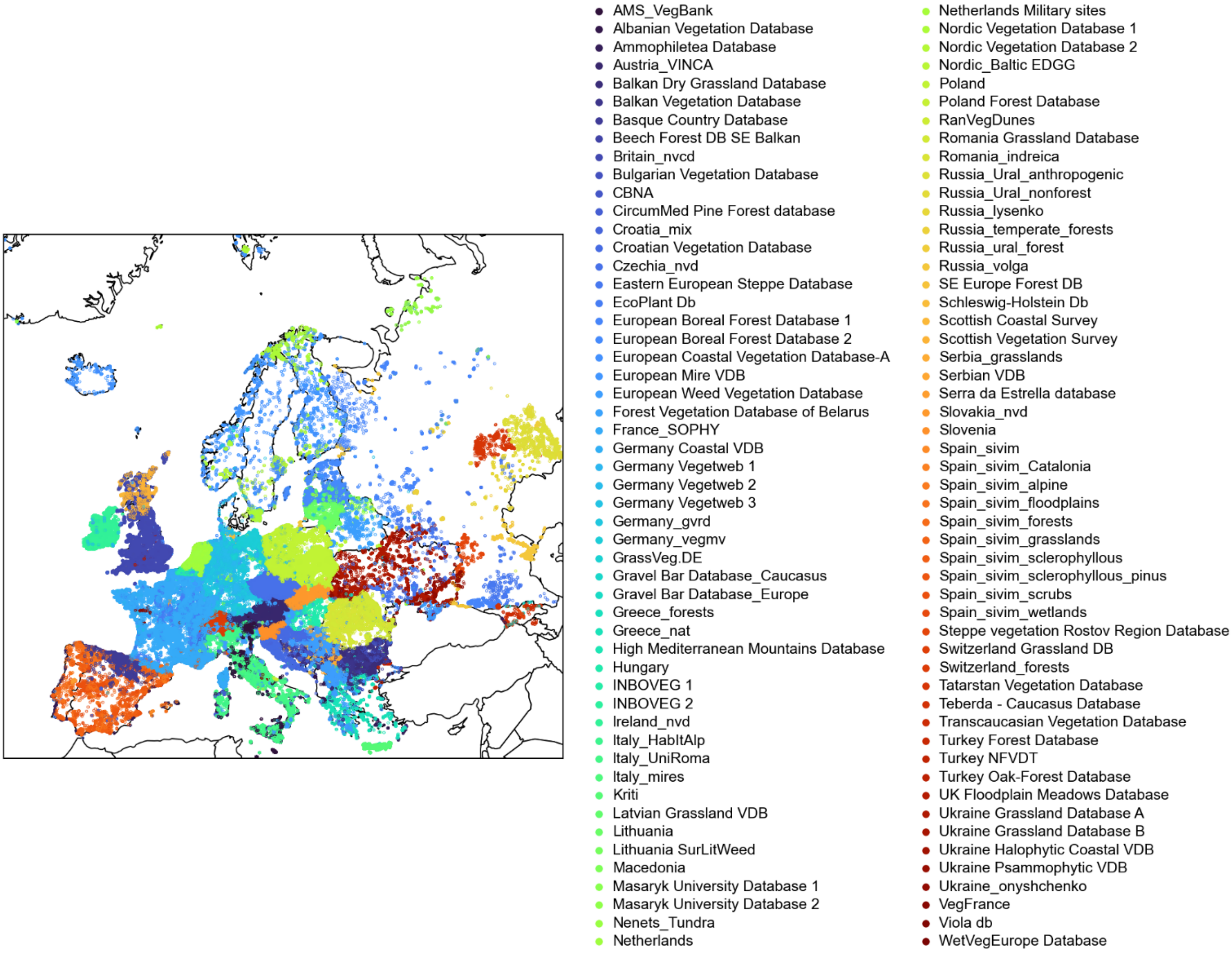

Supplementary Fig. 19. Vegetation plots used in this study, colored by database. The dataset includes 819,232 individual plots of the European Vegetation Archive. See Supplementary Table 4 for the number of plots by database.

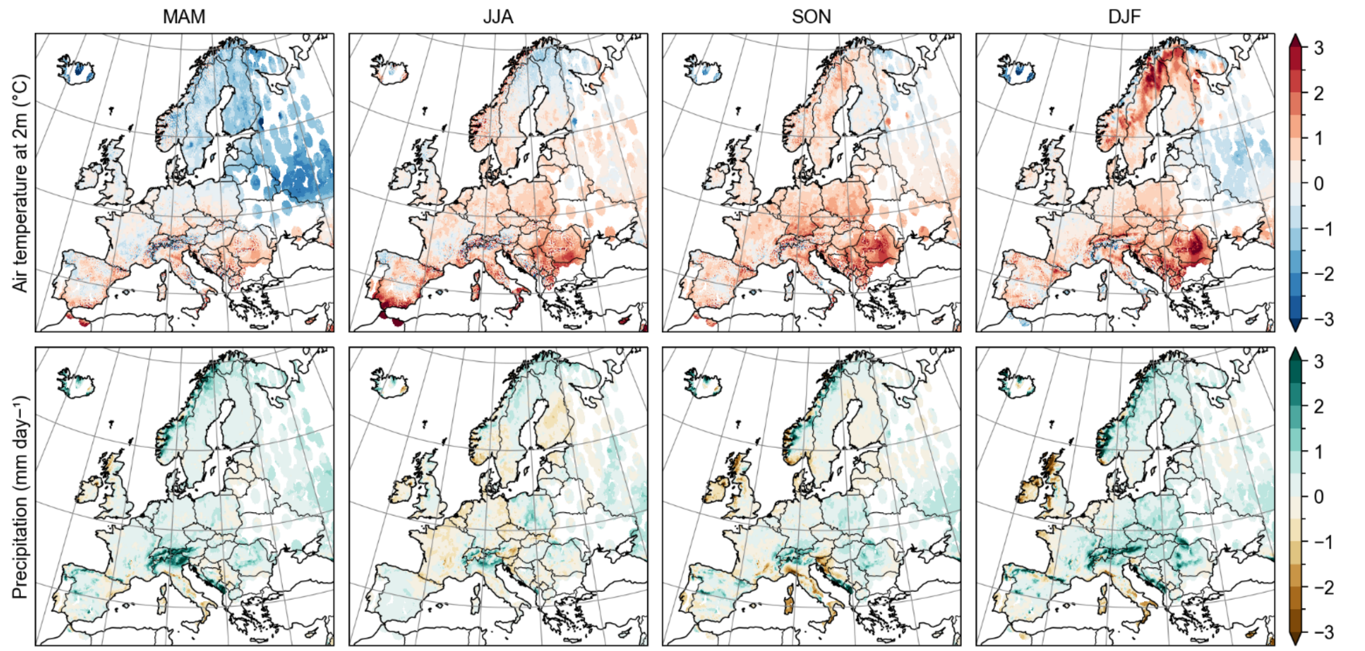

Supplementary Fig. 20. Bias in COSMO-CLM². Model bias indicated by the difference in seasonal climatologies (2011-2015) of 2 m air temperature and precipitation, comparing COSMO-CLM² with E-OBS. MAM = March, April, May; JJA = June, July, August; SON = September, October, November; DJF = December, January, February.

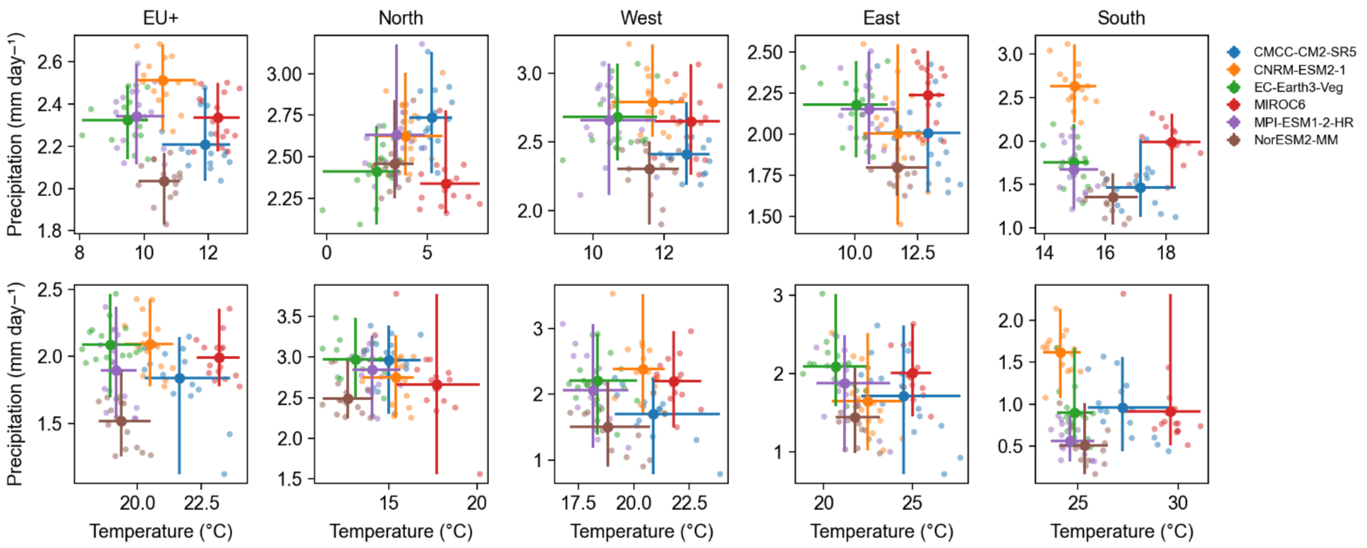

Supplementary Fig. 21. Comparison of MPI-ESM1-2-HR with other CMIP6 models. Annual mean (top row) and summer mean (bottom row) temperature and precipitation as simulated by six global climate models from the CMIP6 generation over the European domain under the SSP1-2.6 scenario. Large dots indicate the mean across 2036-2050, small dots the individual years, and error bars extend from the lowest to the highest annual value.

Supplementary Tables

Supplementary Table 1. Changes in plant functional type cover (Mha and percent). Changes are provided for the scenarios Nature for Nature (NfN), Nature for Society (NfS), and Nature as Culture (NaC) relative to the reference (SSP1), for the EU+ region and the subregions. Note the differences in total area and thus in the percentages. 1 Mh corresponds to 0.01 km^2 10^6. Changes are calculated as net transitions at the grid cell level and then aggregated, such that opposing transitions cancel within grid cells but not between grid cells.

Region	NfN-SSP1	NfS-SSP1	NaC-SSP1	Total area
EU+	48.7 (10.0%)	51.1 (10.5%)	102.6 (21.0%)	488.0
North	5.5 (5.1%)	5.6 (5.1%)	9.2 (8.5%)	108.9
West	18.6 (13.9%)	19.1 (14.3%)	33.5 (25.0%)	133.8
East	18.9 (14.4%)	19.3 (14.7%)	33.7 (25.6%)	131.4
South	4.0 (4.2%)	5.1 (5.5%)	21.3 (22.7%)	93.8

Supplementary Table 2. Environmental predictors used to model and map the distribution of EUNIS level 3 habitats at 1 km resolution. Short descriptions and data sources are provided for each predictor. EUNIS = European Nature Information System.

	Predictor	Data source
Climate	Annual mean temperature (°C)<br>Temperature seasonality<br>Growing degree days heat sum above 5°C (°C)<br>Yearly total precipitation (kg m^{-2} yr^{-1})<br>Precipitation seasonality<br>Snow covered days (day count)<br>Snow water equivalent (kg m^{-2} yr^{-1})	CHELSA V2.1 (1 km resolution)<br>Temporal range: 1981-2010<br>Spatial coverage: Europe
Terrain	EU DEM slope median (degrees)<br>EU DEM aspect northness, eastness (%)<br>Landform classification based on the topographic position index (10 classes)	EU DEM (1 km resolution)<br>EU-DEM (upscaled to 1 km resolution using SAGA-GIS)
Hydrography	Distance to inland water (m)<br>Coastal (binary); coastline defined with a buffer of 5 km around the shapefile	EU-Hydro river network (1 km resolution)<br>EU-Hydro coastline shapefile (1 km resolution)
Geology	Dominant parent material class (27 classes)<br>Depth to rock (m)	European soil database ESDB (1 km resolution)
Soil	Available Water Capacity (AWC) for the topsoil fine earth fraction (-)<br>Bulk density of the topsoil (kg dm^{-3})<br>Coarse fragments (%) content in topsoil<br>Sand, silt, and clay proportions (%)<br>Soil acidity<br>Organic carbon content (g kg^{-1})<br>Nitrogen content (g kg^{-1})<br>Calcium carbonates (g kg^{-1})<br>Cation exchange capacity (cmol kg^{-1})	LUCAS topsoil physical and chemical properties (1 km resolution)

Supplementary Table 3. Area (km²) or number of 1 km pixels per EUNIS habitat type. Results are provided for the reference (SSP1) and the scenarios Nature for Nature (NfN), Nature for Society (NfS), and Nature as Culture (NaC). Each scenario includes 4,942,087 valid pixels in total. Habitat types are aggregated at EUNIS levels 1 and 2. EUNIS = European Nature Information System.

EUNIS level 1	EUNIS level 2	SSP1	NfN	NfS	NaC
MA2	MA21	1998	1998	1998	1998
MA2	MA22	38142	38142	38142	38142
MA2	MA23	4978	4978	4978	4978
MA2	MA24	567	567	567	567
MA2	MA25	3671	3671	3671	3671
N	N1	77130	78408	82657	71485
N	N2	43173	43952	42644	42368
N	N3	30352	30268	28936	27824
P	Pa	19988	19988	19988	19988
P	Pb	1863	1863	1863	1863
P	Pc	32298	32298	32298	32298
P	Pd	5901	5901	5901	5901
P	Pe	1070	1070	1070	1070
P	Pf	38062	38062	38062	38062
P	Pg	21862	21862	21862	21862
P	Ph	27639	27639	27639	27639
P	Pi	1731	1731	1731	1731
P	Pj	2255	2255	2255	2255
Q	Q1	92258	92258	92258	92258
Q	Q2	40641	40641	40641	40641
Q	Q4	93393	93393	93393	93393
Q	Q5	51261	51261	51261	51261
Q	Q6	3658	3658	3658	3658
R	R1	150857	185734	181522	195076
R	R2	35066	37193	31305	36809
R	R3	114401	176894	158553	168936
R	R4	37159	29948	35254	30386
R	R5	84518	90190	75740	88878
R	R6	4803	12883	6768	5578
R	R7	5049	7110	7015	7178
S	S1	26016	37695	34717	36547
S	S2	40825	47094	44386	45927
S	S3	32874	30332	21940	23521
S	S4	16047	12002	8113	10615
S	S5	22287	17992	12224	11602
S	S6	27027	20818	12299	13266
S	S7	11489	9321	8279	8371
S	S9	6576	6475	3026	3849
T	T1	857023	733405	734510	707527
T	T2	79025	77102	81566	78107
T	T3	767827	725961	730344	719918
U	U2	26484	26997	27042	27039
U	U3	17892	18015	18311	18407
U	U4	7519	7519	7519	7519
U	U6	493	496	496	496
U	U7	1341	1352	1434	1405
V	V1	1247453	1365113	1398885	1457575
V	V3	412948	326744	345628	312395
V	V5	183188	210686	212511	202896
URBAN	URBAN	92009	91152	105227	97351

Supplementary Table 4. Number of vegetation plots per database within the European Vegetation Archive (EVA) used to decompose the EUNIS level 3 habitat types into plant functional types. EUNIS = European Nature Information System.

Database	Number of plots
AMS_VegBank	7684
Albanian Vegetation Database	300
Ammophiletea Database	6802
Austria_VINCA	32064
Balkan Dry Grassland Database	5179
Balkan Vegetation Database	10282
Basque Country Database	12587
Beech Forest DB SE Balkan	1042
Britain_nvcd	22190
Bulgarian Vegetation Database	1151
CBNA	2650
CircumMed Pine Forest database	1925
Croatia_mix	1602
Croatian Vegetation Database	10114
Czechia_nvd	75718
Eastern European Steppe Database	5504
EcoPlant Db	3361
European Boreal Forest Database 1	2764
European Boreal Forest Database 2	4140
European Coastal Vegetation Database-A	3587
European Mire VDB	9839
European Weed Vegetation Database	9227
Forest Vegetation Database of Belarus	772
France_SOPHY	106934
Germany Coastal VDB	27
Germany Vegetweb 1	10532
Germany Vegetweb 2	7352
Germany Vegetweb 3	1518
Germany_gvrd	26226
Germany_vegmv	30777
GrassVeg.DE	6665
Gravel Bar Database_Caucasus	65
Gravel Bar Database_Europe	695
Greece_forests	513
Greece_nat	3406
High Mediterranean Mountains Database	579
Hungary	3698
INBOVEG 1	3842
INBOVEG 2	684
Ireland_nvd	14951
Italy_HabItAlp	3195
Italy_UniRoma	22776
Italy_mires	718
Kriti	3729
Latvian Grassland VDB	4382
Lithuania	5926
Lithuania SurLitWeed	1116
Macedonia	270
Masaryk University Database 1	1477
Masaryk University Database 2	356
Nenets_Tundra	844
Netherlands	85489
Netherlands Military sites	7847
Nordic Vegetation Database 1	4299
Nordic Vegetation Database 2	555
Nordic_Baltic EDGG	1885
Poland	60250

Poland Forest Database	2608
RanVegDunes	1776
Romania Grassland Database	16636
Romania_indreica	4628
Russia_Ural_anthropogenic	3109
Russia_Ural_nonforest	2126
Russia_lysenko	737
Russia_temperate_forests	2246
Russia_ural_forest	167
Russia_volga	9235
SE Europe Forest DB	3028
Schleswig-Holstein Db	1630
Scottish Coastal Survey	4864
Scottish Vegetation Survey	4508
Serbia_grasslands	3511
Serbian VDB	824
Serra da Estrella database	813
Slovakia_nvd	28796
Slovenia	14358
Spain_sivim	1232
Spain_sivim_Catalonia	244
Spain_sivim_alpine	2115
Spain_sivim_floodplains	1715
Spain_sivim_forests	1834
Spain_sivim_grasslands	4075
Spain_sivim_sclerophyllous	1014
Spain_sivim_sclerophyllous_pinus	1892
Spain_sivim_scrubs	1631
Spain_sivim_wetlands	2406
Steppe vegetation Rostov Region Database	959
Switzerland Grassland DB	4039
Switzerland_forests	10094
Tatarstan Vegetation Database	2548
Teberda - Caucasus Database	659
Transcaucasian Vegetation Database	260
Turkey Forest Database	81
Turkey NFVDT	234
Turkey Oak-Forest Database	34
UK Floodplain Meadows Database	10636
Ukraine Grassland Database A	6193
Ukraine Grassland Database B	279
Ukraine Halophytic Coastal VDB	647
Ukraine Psammophytic VDB	578
Ukraine_onyshchenko	2276
VegFrance	1068
Viola db	821
WetVegEurope Database	6
Total	819232

Supplementary Table 5. Land cover composition (% area) in the EU+ region. Results are provided for the reference (SSP1) and the scenarios Nature for Nature (NfN), Nature for Society (NfS), and Nature as Culture (NaC). Recent land cover is shown for comparison, using either the habitat mapping and PFT translation of this study for land systems in 2015, or the original CLM5 land surface characterisation based on satellite-observed land cover in 2005. The land cover categories aggregate several plant functional types (PFTs): tree needleleaf includes evergreen temperate, evergreen boreal, and deciduous boreal; tree broadleaf includes evergreen temperate and deciduous temperate; shrub includes broadleaf evergreen temperate, broadleaf deciduous temperate, and broadleaf deciduous boreal; grass includes C3 arctic, C3, and C4; and crop includes rainfed and irrigated. Tropical PFT variants are not present.

Land cover	SSP1	NfN	NfS	NaC	Recent	Recent (CLM5)
Tree needleleaf	8.5	7.8	7.7	8.0	7.8	20.4
Tree broadleaf	13.4	11.4	11.1	11.7	10.5	8.3
Shrub	5.6	5.3	4.9	5.8	4.4	2.2
Grass	26.5	28.1	27.6	39.3	29.6	39.1
Crop	36.3	37.5	38.8	24.2	38.1	24.2
Bare	4.9	5.2	5.1	6.2	4.9	0.8

Supplementary Table 6. Bias in COSMO-CLM^2. Model bias indicated by the root mean square error (RMSE) and mean absolute error (MAE) over the European domain, comparing COSMO-CLM^2 with E-OBS in different seasons (MAM = March, April, May; JJA = June, July, August; SON = September, October, November; DJF = December, January, February).

	2 m air temperature (°C)		Precipitation (mm day^{-1})	
	RMSE	MAE	RMSE	MAE
MAM	1.04	0.82	0.68	0.45
JJA	0.86	0.61	0.50	0.38
SON	0.82	0.63	0.72	0.45
DJF	0.97	0.72	0.85	0.55

Supplementary References

1 Si-Moussi, S. & Thuiller, W. (2025). *Current and future scenarios of EUNIS habitats distribution in Europe (1.0.0).* https://doi.org/10.5281/zenodo.15307415

2 Dou, Y., Zagaria, C., O'Connor, L., Thuiller, W. & Verburg, P. H. Using the Nature Futures Framework as a lens for developing plural land use scenarios for Europe for 2050. *Global Environmental Change* **83**, 102766 (2023). https://doi.org/10.1016/j.gloenvcha.2023.102766

3 EEA. *EUNIS terrestrial habitat classification 2021*, <https://www.eea.europa.eu/data-and-maps/data/eunis-habitat-classification-1> (2021).

4 Chytrý, M. *et al.* European Vegetation Archive (EVA): an integrated database of European vegetation plots. *Applied Vegetation Science* **19**, 173–180 (2016). https://doi.org/10.1111/avsc.12191

5 Chytrý, M. *et al.* (2021). *EUNIS-ESy: Expert system for automatic classification of European vegetation plots to EUNIS habitats (v2021-06-01).* Zenodo. https://doi.org/10.5281/zenodo.4812736

6 Chytrý, M. *et al.* EUNIS Habitat Classification: Expert system, characteristic species combinations and distribution maps of European habitats. *Applied Vegetation Science* **23**, 648–675 (2020). https://doi.org/10.1111/avsc.12519

7 Lawrence, D. M. *et al. Technical Description of version 5.0 of the Community Land Model (CLM)*, <https://escomp.github.io/CTSM/release-clm5.0/index.html> (2018).

8 EEA. *Land cover and change accounts 2000-2018 derived from the CORINE Land Cover 2018*, <https://www.eea.europa.eu/en/analysis/maps-and-charts/land-cover-and-change-statistics-dashboards> (2019).

9 Poulter, B. *et al.* Plant functional type mapping for earth system models. *Geosci. Model Dev.* **4**, 993–1010 (2011). https://doi.org/10.5194/gmd-4-993-2011

10 Poulter, B. *et al.* Plant functional type classification for earth system models: results from the European Space Agency's Land Cover Climate Change Initiative. *Geosci. Model Dev.* **8**, 2315–2328 (2015). https://doi.org/10.5194/gmd-8-2315-2015

11 Schupfner, M. *et al.* (2020). *CMIP6 ScenarioMIP DKRZ MPI-ESM1-2-HR ssp126_r1i1p1f1 - RCM-forcing data.* World Data Center for Climate (WDCC) at DKRZ. https://doi.org/10.26050/WDCC/RCM_CMIP6_SSP126-HR_r1i1p1f1
12 Merrifield, A. L., Brunner, L., Lorenz, R., Humphrey, V. & Knutti, R. Climate model Selection by Independence, Performance, and Spread (ClimSIPS v1.0.1) for regional applications. *Geosci. Model Dev.* **16**, 4715–4747 (2023). https://doi.org/10.5194/gmd-16-4715-2023
13 Sobolowski, S. *et al.* EURO-CORDEX GCM Selection & Ensemble Design: Implementation Framework. (Zenodo, 2023). https://doi.org/10.5281/zenodo.7673400
14 Cornes, R. C., van der Schrier, G., van den Besselaar, E. J. M. & Jones, P. D. An Ensemble Version of the E-OBS Temperature and Precipitation Data Sets. *Journal of Geophysical Research: Atmospheres* **123**, 9391–9409 (2018). https://doi.org/10.1029/2017JD028200
15 Muñoz Sabater, J. (2019). *ERA5-Land hourly data from 1950 to present.* Copernicus Climate Change Service (C3S) Climate Data Store (CDS). https://doi.org/10.24381/cds.e2161bac
