# Supplementary material for: Climate response to Nature Future scenarios in a regional Earth System Model
Source: Nat Commun. 2026 Mar 16;17:4017. doi: 10.1038/s41467-026-70284-8 (PMC13136345; doi:10.1038/s41467-026-70284-8)
Supplement: Supplementary file 3 — Description of Additional Supplementary Files [file 41467_2026_70284_MOESM3_ESM.pdf]

Description of Additional Supplementary Files

File Name: Supplementary Data 1
Description: **Crosswalk rules used to convert the land system in each pixel to a EUNIS level 1 group.** Crosswalk rules and land systems' composition (% area) of habitats at EUNIS level 3. EUNIS = European Nature Information System.

File Name: Supplementary Data 2
Description: **Average cover (% area) of individual plant functional types for 235 EUNIS level 3 habitat types.** Averages are calculated based on all plots of the European Vegetation Archive (EVA) classified as belonging to the same habitat type. EUNIS = European Nature Information System.

File Name: Supplementary Data 3
Description: **Climate response to Nature Future scenarios.** Climate (2036-2050 mean ± one standard deviation across years) in the reference (SSP1) and responses in the scenarios Nature for Nature (NfN), Nature for Society (NfS), and Nature as Culture (NaC) relative to SSP1. Annual means and annual maximum temperature, and seasonal means for air temperature at 2 m, soil temperature at 0-10 cm, wind speed at 10 m, precipitation, soil moisture at 0-10 cm, and gross primary production (GPP). Seasons are defined as MAM = March, April, May; JJA = June, July, August; SON = September, October, November; and DJF = December, January, February. Results are summarised for the EU+ region, the four European subregions, the 1% most affected areas per scenario, the 1% most extreme areas in SSP1 (e.g., hottest areas), and the 1% most changing areas in SSP1 compared with recent climate (e.g., most heating areas). For reference, results are provided for the recent historical period (2006-2015) and the difference between SSP1 and recent conditions. Statistical significance of the climate response per scenario (i.e., scenario minus SSP1) is based on a two-sided Wilcoxon signed-rank test. Statistical significance of changes in SSP1 compared with recent climate (i.e., SSP1 minus Recent) is based on a two-sided Mann-Whitney U rank test.
