# Supplementary material for: Climate response to Nature Future scenarios in a regional Earth System Model
Source: Nat Commun. 2026 Mar 16;17:4017. doi: 10.1038/s41467-026-70284-8 (PMC13136345; doi:10.1038/s41467-026-70284-8)
Supplement: Supplementary file 7 — Reporting Summary [file 41467_2026_70284_MOESM7_ESM.pdf]

nature portfolio

Corresponding author(s): Petra Sieber

Last updated by author(s): 06/02/2026

Reporting Summary

Nature Portfolio wishes to improve the reproducibility of the work that we publish. This form provides structure for consistency and transparency in reporting. For further information on Nature Portfolio policies, see our Editorial Policies and the Editorial Policy Checklist.

Statistics

For all statistical analyses, confirm that the following items are present in the figure legend, table legend, main text, or Methods section.

n/a	Confirmed	
[ ]	[x]	The exact sample size (n) for each experimental group/condition, given as a discrete number and unit of measurement
[x]	[ ]	A statement on whether measurements were taken from distinct samples or whether the same sample was measured repeatedly
[ ]	[x]	The statistical test(s) used AND whether they are one- or two-sided<br>*Only common tests should be described solely by name; describe more complex techniques in the Methods section.*
[ ]	[x]	A description of all covariates tested
[ ]	[x]	A description of any assumptions or corrections, such as tests of normality and adjustment for multiple comparisons
[ ]	[x]	A full description of the statistical parameters including central tendency (e.g. means) or other basic estimates (e.g. regression coefficient) AND variation (e.g. standard deviation) or associated estimates of uncertainty (e.g. confidence intervals)
[ ]	[x]	For null hypothesis testing, the test statistic (e.g. F, t, r) with confidence intervals, effect sizes, degrees of freedom and P value noted<br>*Give P values as exact values whenever suitable.*
[x]	[ ]	For Bayesian analysis, information on the choice of priors and Markov chain Monte Carlo settings
[x]	[ ]	For hierarchical and complex designs, identification of the appropriate level for tests and full reporting of outcomes
[ ]	[x]	Estimates of effect sizes (e.g. Cohen's d, Pearson's r), indicating how they were calculated

Our web collection on statistics for biologists contains articles on many of the points above.

Software and code

Policy information about availability of computer code

Data collection	The ESM data generated in this study have been deposited in the ETH Research Collection at https://doi.org/10.3929/ethz-c-000795598. The land system scenarios used in this study are available at https://doi.org/10.34894/NWGCBY. The habitat projections used in this study are available at https://doi.org/10.5281/zenodo.15307415. The raw vegetation plot data belong to the owners or contributors of each vegetation database and can be requested at https://euroveg.org/eva-database/obtaining-data.
Data analysis	The code used to generate the presented data and figures is available at https://github.com/pesieber/Sieber-etal-2026_NCOMMS.git. The translation of EUNIS habitats to PFTs is available at https://gitlabext.wsl.ch/karger/eunis2pft The COSMO-CLM2 regional ESM consists of three components. The source code of COSMO-Model 6 is available to research institutions free of charge under an institutional license from https://www.cosmo-model.org/content/default.htm (last access: October 2022). CLM5 is a publicly released version of the Community Land Model available from https://github.com/ESCOMP/CTSM (last access: October 2022). The OASIS coupler is a community software available from https://oasis.cerfacs.fr/en/home/ (last access: January 2020).

For manuscripts utilizing custom algorithms or software that are central to the research but not yet described in published literature, software must be made available to editors and reviewers. We strongly encourage code deposition in a community repository (e.g. GitHub). See the Nature Portfolio guidelines for submitting code & software for further information.

Data

Policy information about availability of data

All manuscripts must include a data availability statement. This statement should provide the following information, where applicable:

- Accession codes, unique identifiers, or web links for publicly available datasets
- A description of any restrictions on data availability
- For clinical datasets or third party data, please ensure that the statement adheres to our policy

ESM data presented in this paper will be available from the ETH Research Collection after acceptance. Original vegetation plot data belong to the owners or contributors of each vegetation database and can be requested at https://euroveg.org/eva-database/obtaining-data.

Research involving human participants, their data, or biological material

Policy information about studies with human participants or human data. See also policy information about sex, gender (identity/presentation), and sexual orientation and race, ethnicity and racism.

Reporting on sex and gender	*Use the terms sex (biological attribute) and gender (shaped by social and cultural circumstances) carefully in order to avoid confusing both terms. Indicate if findings apply to only one sex or gender; describe whether sex and gender were considered in study design; whether sex and/or gender was determined based on self-reporting or assigned and methods used.*<br>*Provide in the source data disaggregated sex and gender data, where this information has been collected, and if consent has been obtained for sharing of individual-level data; provide overall numbers in this Reporting Summary. Please state if this information has not been collected.*<br>*Report sex- and gender-based analyses where performed, justify reasons for lack of sex- and gender-based analysis.*
Reporting on race, ethnicity, or other socially relevant groupings	*Please specify the socially constructed or socially relevant categorization variable(s) used in your manuscript and explain why they were used. Please note that such variables should not be used as proxies for other socially constructed/relevant variables (for example, race or ethnicity should not be used as a proxy for socioeconomic status).*<br>*Provide clear definitions of the relevant terms used, how they were provided (by the participants/respondents, the researchers, or third parties), and the method(s) used to classify people into the different categories (e.g. self-report, census or administrative data, social media data, etc.)*<br>*Please provide details about how you controlled for confounding variables in your analyses.*
Population characteristics	*Describe the covariate-relevant population characteristics of the human research participants (e.g. age, genotypic information, past and current diagnosis and treatment categories). If you filled out the behavioural & social sciences study design questions and have nothing to add here, write "See above."*
Recruitment	*Describe how participants were recruited. Outline any potential self-selection bias or other biases that may be present and how these are likely to impact results.*
Ethics oversight	*Identify the organization(s) that approved the study protocol.*

Note that full information on the approval of the study protocol must also be provided in the manuscript.

Field-specific reporting

Please select the one below that is the best fit for your research. If you are not sure, read the appropriate sections before making your selection.

☐ Life sciences ☐ Behavioural & social sciences ☒ Ecological, evolutionary & environmental sciences

For a reference copy of the document with all sections, see nature.com/documents/nr-reporting-summary-flat.pdf

Ecological, evolutionary & environmental sciences study design

All studies must disclose on these points even when the disclosure is negative.

Study description	In this study, we explore how nature management in Europe would affect European climate mid-century. To this end, we implement three nature management scenarios and one reference scenario in a regional Earth System Model. The scenarios are based on detailed land use, habitat, and species projections. The regional Earth System Model simulates responses in land-atmosphere exchanges and key climate variables like temperature, precipitation, or gross primary production.
Research sample	Each of the four scenarios is simulated for 15 years (excluding a 2-year spin up period). Annual and seasonal averages are calculated as the mean of 2036-2050, and the standard deviation is calculated across years. The period was chosen to represent mid-century conditions under a low-emission scenario (SSP1-2.6).
Sampling strategy	No sampling was performed.
Data collection	We use output of the regional Earth System Model. PS performed the simulations, postprocessing, and analysis.

Timing and spatial scale	Each scenario was simulated at 11° (~12.5 km) resolution for the period 2036-2050, with monthly, daily, and partly sub-daily (e.g. daily maxima) outputs. The period was chosen to represent mid-century conditions.
Data exclusions	Data from the 2-year spin-up period was excluded.
Reproducibility	The simulations are reproducible based on the provided methods.
Randomization	Not applicable.
Blinding	Not applicable.

Did the study involve field work? ☐ Yes ☒ No

Reporting for specific materials, systems and methods

We require information from authors about some types of materials, experimental systems and methods used in many studies. Here, indicate whether each material, system or method listed is relevant to your study. If you are not sure if a list item applies to your research, read the appropriate section before selecting a response.

Materials & experimental systems

n/a	Involved in the study
☒	☐ Antibodies
☒	☐ Eukaryotic cell lines
☒	☐ Palaeontology and archaeology
☒	☐ Animals and other organisms
☒	☐ Clinical data
☒	☐ Dual use research of concern
☒	☐ Plants

Methods

n/a	Involved in the study
☒	☐ ChIP-seq
☒	☐ Flow cytometry
☒	☐ MRI-based neuroimaging

Plants

Seed stocks	na
Novel plant genotypes	na
Authentication	na
